# Social mobility across the lifecourse and DNA methylation age acceleration in adults in the UK

**DOI:** 10.1038/s41598-022-26433-2

**Published:** 2022-12-24

**Authors:** Yanchun Bao, Tyler Gorrie-Stone, Eilis Hannon, Amanda Hughes, Alexandria Andrayas, Grant Neilson, Joe Burrage, Jonathon Mill, Leonard Schalkwyk, Meena Kumari

**Affiliations:** 1 Department of Mathematical Sciences, University of Essex, Colchester, UK; 2 Institute for Social and Economic Research, University of Essex, Wivenhoe Park, Colchester, UK; 3 School of Life Sciences, University of Essex, Colchester, UK; 4 Diamond Light Source, Didcot, UK; 5 Department of Clinical and Biomedical Sciences, Faculty of Health and Life Sciences, University of Exeter Medical School, University of Exeter, Exeter, UK; 6 Integrative Epidemiology Unit, University of Bristol, Bristol, UK

**Keywords:** Epigenetics, Epidemiology

## Abstract

Disadvantaged socio-economic position (SEP) is associated with greater biological age, relative to chronological age, measured by DNA methylation (positive ‘age acceleration’, AA). Social mobility has been proposed to ameliorate health inequalities. This study aimed to understand the association of social mobility with positive AA. Diagonal reference modelling and ordinary least square regression techniques were applied to explore social mobility and four measures of age acceleration (first-generation: ‘Horvath’, ‘Hannum’ and second-generation: ‘Phenoage’, DunedinPoAm) in n = 3140 participants of the UK Household Longitudinal Study. Disadvantaged SEP in early life is associated with positive AA for three (Hannum, Phenoage and DunedinPoAm) of the four measures examined while the second generation biomarkers are associated with SEP in adulthood (p < 0.01). Social mobility was associated with AA measured with Hannum only such that compared to no mobility, upward mobility was associated with greater age independently of origin and destination SEP. Compared to continuously advantaged groups, downward mobility was associated with positive Phenoage (1.06y [− 0.03, 2.14]) and DunedinPoAm assessed AA (0.96y [0.24, 1.68]). For these two measures, upward mobility was associated with negative AA (Phenoage, − 0.65y [− 1.30, − 0.002]; DunedinPoAm, − 0.96y [− 1.47, − 0.46]) compared to continually disadvantaged groups. While we find some support for three models of lifecourse epidemiology with early life as a sensitive period, SEP across the lifecourse and social mobility for age acceleration measured with DNA methylation, our findings suggest that disadvantaged SEP across the lifecourse is most consistently associated with positive AA.

## Introduction

The association between unfavourable socioeconomic circumstances in childhood and adulthood and increased morbidity and mortality is a near ubiquitous observation^1–4^. Three models have been proposed to explain the link between life course socio-economic position (SEP) and adult disease^5^. The critical/sensitive period model proposes that adverse early life SEP can exert permanent changes to biology with long-term health effects. Thus, socioeconomic origins exert significant and independent effects on later life health, reflecting the “long arm” of childhood circumstances^6,7^. The accumulation model posits accumulation of disadvantage across the life course and negative impacts on biology and health. Thus, the consequences of cumulative advantage and disadvantage, whereby stresses and strains accrue over the life course to a greater extent among those in disadvantaged SEP, set in motion more rapid ageing or weathering of biological systems under conditions of chronic adversity^8–10^. Social mobility frames SEP changes across the life course with differential association of stable or varying SEP trajectories on biology and health. An unresolved question in social epidemiological research is whether the movement between origin and destination SEPs, per se, influences health net of origin and destination effects.

Social mobility may constrain SEP differences in health such that upward social mobility (i.e., individuals’ rising in the SEP hierarchy) would be associated with improved health compared to the situation of stable disadvantaged SEP (i.e., remaining in low SEP over time). In turn, downward social mobility (i.e., deteriorating in SEP hierarchy) is considered to have negative effects on health compared to the situation of stable advantaged SEP (i.e., always experiencing advantaged SEP over time)^11,12^. Social mobility is dependent on both social origin and destination and traditional regression frameworks should separately estimate the effects of socioeconomic origin, destination, and social mobility simultaneously. However, this is not typically assessed^13^. An example of an approach to overcome these methodological limitations is Sobel’s diagonal reference model^14^.

Rapid ageing, often calculated as a greater biological than chronological age can be measured in a number of ways, for example methods have been developed using DNA methylation. This is the most widely studied epigenetic modification in relation to human health and disease and “epigenetic clocks”, including those by Horvath^15^ and Hannum et al.^16^, have been developed that predict age from DNA methylation (DNAm) profiles. Although DNAm age and chronological age are highly correlated, the relationship varies between individuals, such that some people predicted based on their DNA methylation profile as “older” than their chronological age and others are predicted as “younger”. DNAm age older than chronological age, so called ‘age acceleration’ or positive age acceleration where DNAm age is greater than chronological age, has been associated with mortality and morbidity independently of age^17^. Recently ‘second generation’ DNAm age algorithms, for example, the ‘Phenoage’ or Levine algorithm^18^ and ‘DunedinPoAm’^19^ have been described. These algorithms are estimated with chronological age and phenotypic aging measures which consists of clinical biomarkers and are considered to more closely represent biological age than the first-generation algorithms.

We have previously demonstrated that positive age acceleration, measured by means of the first generation algorithms, is associated with childhood social class and educational attainment but not with measures of social position later in life. These observations, while not universal^20^, suggest a lasting influence of early-life conditions on positive age acceleration^21^. Others have described positive age acceleration in groups that were persistently disadvantaged across the lifecourse compared to those that were persistently advantaged, with the upwardly and downwardly mobile groups ranking somewhere in between. However, this latter observation was not significant^22,23^ and it is unclear whether this is due to the inability of the analytic approach used to examine mobility separately from early life and later SEP. Recent studies suggest that second generation algorithms are more closely related to socio-economic conditions than first generation algorithms^24^, which may be important as they more closely reflect biological age.

Here we examine the association of social mobility with age acceleration, which is defined as the residuals of the linear regression of epigenetic age on chronological age^25^ in a UK population. We expand our earlier analyses in a number of ways: we use diagonal reference model (DRM) to assess the association of social mobility with DNAm age. We examine the relative contribution of early life social position and adult social position measured by highest household occupation at aged 14 and adulthood. Further, we examine the association with the second-generation algorithms, ‘phenoage’ and ‘DunedinPoAm’.

## Methods

### Participants

*Understanding Society* the UK Household Longitudinal Study (UKHLS) is a nationally representative study which started in 2009 aiming to recruit individuals in 25,000 households^26^. Specifically, this project is based on data from the British Household Panel Survey (BHPS) and a subsample of the General Population Sample (GPS) samples in waves 3 and 2 (2010–2012) respectively. Details of the blood sample collection can be found elsewhere^27^. All methods were carried out in accordance with Information Commissioner’s Office guidelines and regulations. Informed consent was obtained from all participants and/or their legal guardian(s). The University of Essex Ethics Committee has approved all data collection on *Understanding Society* main study. Approval for asking for consent and the collection of biosocial data by trained nurses in Waves 2 and 3 of the main survey was obtained from the National Research Ethics Service (Understanding Society—UK Household Longitudinal Study: A Biosocial Component, Oxfordshire A REC, Reference: 10/H0604/2).

Due to the availability of funding, methylation was profiled at two different time points: in 2017 from DNA extracted from whole blood from 1193 persons and in 2020 from DNA extracted from whole blood from a further 2536 resulting in a total n = 3728 who were eligible for and consented to blood sampling and genomic analyses. Eligibility requirements for genetic analyses meant that the methylation sample was restricted to participants who reported their ethnicity as White/European.

### DNA methylation

Five hundred–nanogram samples of whole-blood DNA from 1193 and 2536 persons were treated with sodium bisulfite using the EZ96 DNA methylation kit (Zymo Research, Irvine, California) following the manufacturer’s standard protocol. DNA methylation was assessed using the Illumina Infinium HumanMethylationEPIC BeadChip kit (Illumina, Inc., San Diego, California). DNA methylation levels were quantified on an Illumina HiScan System (Illumina, Inc.). Raw signal intensities were parsed into R (R Foundation for Statistical Computing, Vienna, Austria) and converted into β values using the Bioconductor watermelon and bigmelon package^28^. Outliers were identified and removed using ‘wateRmelon::outlyx’. Low quality samples (< 85% bisulfite conversion) identified and removed using ‘wateRmelon::bscon’. Data was then normalised using ‘wateRmelon::dasen’, difference between normalised and raw data per sample estimated using ‘wateRmelon::qual’. Samples were removed on the basis of having a root mean square difference and standard deviation of difference > 0.05. After removal of outlying/poor quality samples from raw data, data was subjected to ‘wateRmelon::pfilter’ and then renormalised using ‘wateRmelon::dasen’. These steps result in 1174 samples and 857,071 probes remaining for the 2017 dataset and 2480 samples and 860,950 remaining in the 2020 dataset. The total number with DNAm measurement is 3654.

### DNA methylation age and age acceleration

DNA methylation (DNAm) age was calculated through linear functions using wateRmelon::agep, supplying different sets of coefficients for Horvath^15^, Hannum^16^ or Phenoage^18^ calculations following

$${DNAm\, age}_{j}=intercept+\sum_{i=1}^{m}{\beta }_{ij}\times {Coef}_{i}, j=1, 2, \dots , n,$$

where *m* is the number of probes and n is the sample size. ${Coef}_{i}$ is the coefficient of $i$th probe and ${\beta }_{ij}$ is the measurement for $i$th probe and $j$th individual $i=\mathrm{1,2}, \dots , m; j=1, 2, \dots n$. Because DNAm age were designed for an earlier microarray measurement, missing probes (20 for Horvath, 7 for Hannum; and 2 for Phenoage, listed in Supplement Table S1 were not included in calculations. DunedinPoAm was calculated from the using information contained within Belsky et al.^19^ using the code in https://github.com/DLCorcoran/DunedinPoAm38.

The age acceleration by each Horvath, Hannum, Phenoage and DunedinPoAm algorithm was then calculated as residuals of linear regression model of each of DNAm age with chronological age and was included in the DRM model as an outcome. A positive value represents positive age acceleration and vice versa. The linear regression model also adjusted for batch effect, using plate as a categorical variable, and white blood cell composition estimates, which were calculated using the Houseman reference-based algorithm implemented in the estimateCellCounts function package in minfi^29,30^.

### Social class

Childhood social class was based on parents’ National Statistics Socio-economic Classification (NS-SEC) when participants were 14 years of age and was categorized as disadvantaged (NS-SEC routine/semi-routine), intermediate (NS-SEC lower management & professional/intermediate/small employers & own account/lower supervisory & technical) and advantaged (NS-SEC large employers & higher management/higher professional). The social class of the parent with the highest social class was used where social class was available for more than one parent. Current social class was based on the NS-SEC of the most advantaged person in the household. We excluded young participants (age below 25) from analysis since they are less likely to have reached occupational maturity.

### Covariates

Sex, standardized chronological age, squared standardized age (to capture possible nonlinearity in age-related confounding) are included as covariates. Further sensitive analysis includes marital status and highest education qualification of participants as covariates. Marital status was categorized as single, married/coupled or divorced/separated. Education attainment was categorized as University degree, A-level, GCSE, no qualification.

### Statistical analyses

The mean and standard deviation of age acceleration of four epigenetic measures by participants’ characteristics and the association between each algorithm with each characteristic was examined by either two sample t-test or one-way ANOVA.

A diagonal reference model (DRM) is used to model the social mobility and age acceleration. In a DRM mobile, groups resemble their origin and destination classes using non mobile groups as the benchmark. Thus, DRM assumes that the outcome (age acceleration in this paper) has a single vector of coefficients for origin classes (childhood social position) and destination classes (adulthood social position) for socially immobile groups (reference groups), and outcome of those who are mobile is the function of values of immobile groups with weighting parameters representing the relative importance of the origin and destination classes. Given a two-way contingency table classified by origin and destination classes, then the mean of the off-diagonal cell (represented as ${\mu }_{ij}$) represents the outcome for the mobile groups moving from $i$th origin class to $j$th destination class ($i\ne j$), is the function of the mean outcomes of the diagonal cell (represented as ${\mu }_{ii}$ and ${\mu }_{jj}$) and covariates is written as:

$${\mu }_{ij}=E\left({Y}_{ij}\right)={\beta }_{0}+p{\mu }_{ii}+\left(1-p\right){\mu }_{jj}+{X}_{ij}\beta ,$$

where ${\beta }_{0}$ is the intercept, subscripts i and j represent the social position of origin and destination, respectively and ${\mu }_{ii}=E\left({Y}_{ii}\right)$ is the mean outcome of origin and ${\mu }_{jj}=E\left({Y}_{jj}\right)$ is the mean outcome of destination. The relative importance of the origin classes is represented by $p$. This has a range between 0 and 1, where 0 corresponds to an outcome which is only associated with the destination class and 1 represents an outcome which is only associated with the origin class. ${X}_{ij}$ is a vector of covariates and can include the mobility variables (for example, mobile vs. immobile contrasts, upward vs. downward contrasts, the number of steps moved though the mobility hierarchy etc.). By including additional social mobility variables in model, DRM can distinguish effects of social mobility itself from the effects of the origin and destination classes. In this paper, for each measure, we consider four models with covariates plus: (1) no mobility variable; (2) any direction mobility variable; (3) variables to indicate upward or downward trend; (4) variables to indicate one step, two step upward or downward trend. Analyses were conducted in STATA, version 16 (StataCorp LP, College Station, Texas) with “drm” package.

### Sensitivity analyses

To understand the robustness of our findings we conduct several sensitivity analyses with estimates provided in supplementary documents. First, socioeconomic and health factors including marital status and highest education qualification were included in analyses. We then repeated analyses with alternate measures of social class using definitions based on mother’s occupation when father’s was not available and ‘own’ occupation rather than the most advantaged occupation in the household. We also stratified analyses by participant median year of birth (born before 1956, n = 1522; and born after 1956, n = 1618) to investigate potential cohort effects. Finally, we investigated the relationship of mobility and age acceleration with two mobile-immobile comparisons: (1) downward mobile group vs immobile socially advantaged group, and (2) upward mobile group vs immobile socially disadvantaged group.

## Results

In total, data from 3140 participants were used in analysis where the mean of participants’ age is 54.5 (standard deviation, 14.0, range 25 to 98). The number and percentage of participants for demographic characteristics are presented in Table 1. With respect to covariates, on average females have negative value of age acceleration, that is three of the four DNA methylation algorithms suggest that they are on average biologically ‘younger’ than chronological age. The DNAm algorithms were not consistently associated with other characteristics, for example with Phenoage and DunedinPoAm, age acceleration was apparent with the covariates divorce, no qualifications, child and adult social class. However, age acceleration indexed with Horvath was only associated with divorce and Hannum was only linearly associated with childhood social class.

**Table 1:** Characteristics of the analytic sample in a study of age acceleration indexed by the epigenetic algorithms Horvath, Hannum, Phenoage and DunedinPoAm: mean and standard deviation (n = 3105) by categorical variables, UK Household Longitudinal Study, 2010–2012.

	N (%)	Mean Horvath (SD)	Mean Hannum (SD)	Mean Phenoage (SD)	Mean DunedinPoAm (SD)	Mean age (SD)
Total	3140 (100)	0.00 (3.75)	0.00 (3.28)	− 0.00 (4.91)	− 0.00 (3.60)	54.5 (14.03)
**Age**						
Old (born before 1956)	1522 (48.5)	0.01 (4.16)	− 0.01 (3.74)	− 0.16 (5.24)	− 0.06 (4.15)	66.6 (7.46)
Young (born in or after 1956)	1618 (51.5)	− 0.01 (3.31)	0.01 (2.79)	0.15 (4.57)	0.06 (2.98)	43.1 (7.89)
Two sample t-test p-value		0.91	0.91	0.08	0.37	< 0.0001
**Sex**						
Male	1388 (44.2)	0.53 (3.79)	0.97 (3.29)	0.05 (4.90)	0.40 (3.54)	55.19 (14.07)
Female	1752 (55.8)	− 0.42 (3.65)	− 0.77 (3.07)	− 0.04 (4.92)	− 0.31 (3.62)	53.93 (13.98)
Two sample t-test p-value		< 0.0001	< 0.0001	0.61	< 0.0001	0.01
**Marital status**						
Single	418 (13.3)	− 0.44 (3.43)	− 0.11 (2.82)	− 0.15 (4.79)	0.33 (2.91)	42.13 (13.02)
Married (coupled)	2036 (64.8)	− 0.003 (3.60)	0.03 (3.16)	− 0.28 (4.60)	− 0.33 (3.48)	55.17 (13.09)
Divorced (separate)	686 (21.9)	0.28 (4.28)	− 0.02 (3.87)	0.94 (5.69)	0.78 (4.14)	59.97 (12.85)
One way ANOVA p-value		0.009	0.73	< 0.0001	< 0.0001	< 0.0001
**Highest education qualification**						
University degree	690 (22.0)	0.12 (3.44)	0.12 (2.92)	− 0.45 (4.51)	− 0.94 (2.80)	49.44 (13.20)
A-level	970 (30.9)	− 0.07 (3.79)	− 0.04 (3.38)	− 0.07 (5.09)	− 0.12 (3.50)	53.13 (13.82)
GCSE	1062 (33.8)	− 0.07 (3.81)	− 0.03 (3.28)	0.10 (4.96)	0.33 (3.60)	54.71 (13.38)
No qualification	409 (13.0)	0.04 (3.88)	− 0.09 (3.63)	0.64 (4.85)	1.02 (4.52)	65.71 (11.20)
Missing data	9 (0.3)	4.01 (5.54)	2.52 (2.24)	2.29 (7.61)	− 0.45 (3.40)	52.22 (9.97)
One way ANOVA p-value		0.69	0.70	0.004	< 0.0001	< 0.0001
**Childhood social class**						
Disadvantage	968 (30.8)	0.13 (3.94)	0.29 (3.34)	0.63 (5.12)	0.46 (3.99)	57. 10 (13.70)
Intermediate	1836 (58.5)	− 0.06 (3.68)	− 0.11 (3.34)	− 0.30 (4.80)	− 0.14 (3.42)	53.90 (14.07)
Advantage	336 (10.7)	− 0.04 (3.49)	− 0.24 (2.70)	− 0.20 (4.72)	− 0.56 (3.19)	50.11 (13.37)
One way ANOVA p-value		0.44	0.003	< 0.0001	< 0.0001	< 0.0001
**Adulthood social class**						
Disadvantage	941 (30.0)	0.05 (3.86)	0.05 (3.40)	0.62 (5.00)	0.81 (3.91)	56.47 (14.30)
Intermediate	776 (24.7)	− 0.11 (3.88)	− 0.38 (3.40)	− 0.22 (5.06)	− 0.08 (3.57)	56.03 (13.84)
Advantage	1423 (45.3)	0.03 (3.59)	0.17 (3.12)	− 0.29 (4.73)	− 0.49 (3.29)	52.33 (13.66)
One way ANOVA p-value		0.62	0.001	< 0.0001	< 0.0001	< 0.0001

Table 2 shows the average age acceleration indexed by Horvath, Hannum, Phenoage and DunedinPoAm, stratified both by origin occupation (row) and destination occupation (column) class. Overall, the arithmetic mean of all four measures is negative for advantaged childhood SEP, that is younger than adult chronological age while this is apparent for second generation measures only in adulthood. The diagonal of the table reveals a social gradient for the socially immobile. Positive age acceleration is observed for stably disadvantaged SEP (Horvath 0.05; Hannum 0.18, Phenoage 0.97 and DunedinPoAm 0.98) indicating DNAm age for this group is higher than expected from their chronological age. On the other hand, mixed results are apparent in those with stable advantage.

**Table 2:** Arithmetic mean of age acceleration indexed by Horvath, Hannum, Phenoage and DunedinPoAm algorithm, by origin (childhood) and destination (adulthood) social class, sample size of each cell is given at the bottom of the table.

Origin class	Destination class			Total
	Horvath			
	Disadvantage	Intermediate	Advantage	
Disadvantage	0.05	0.37	0.06	0.13
Intermediate	0.02	− 0.29	0.01	− 0.06
Advantage	0.38	− 0.55	0.05	− 0.04
Total	0.05	− 0.11	0.03	0.00

Origin class	Hannum			Total
	Disadvantage	Intermediate	Advantage	
Disadvantage	0.18	0.30	0.44	0.29
Intermediate	− 0.05	− 0.64	0.13	− 0.11
Advantage	− 0.15	− 0.90	− 0.03	− 0.24
Total	0.05	− 0.38	0.17	− 0.00

Origin class	Phenoage			Total
	Disadvantage	Intermediate	Advantage	
Disadvantage	0.97	0.67	0.11	0.63
Intermediate	0.29	− 0.76	− 0.36	− 0.30
Advantage	0.70	0.36	− 0.57	− 0.20
Total	0.62	− 0.22	− 0.29	− 0.00

Origin class	DunedinPoAm			Total
	Disadvantage	Intermediate	Advantage	
Disadvantage	0.98	0.59	− 0.37	0.46
Intermediate	0.70	− 0.38	− 0.46	− 0.14
Advantage	0.20	− 0.32	− 0.79	− 0.56
Total	0.81	− 0.08	− 0.49	− 0.00

Origin class	Sample size (n = 3140)			Total
	Disadvantage	Intermediate	Advantage	
Disadvantage	432	234	302	968
Intermediate	467	466	903	1836
Advantage	42	76	218	336
Total	941	776	1423	3140

UK Household Longitudinal Study, 2010–2012.

Tables 3, 4, 5 and 6 present the results for social mobility with age acceleration indexed by Horvath, Hannum, Phenoage and DunedinPoAm, respectively. Table 3 shows that there is no difference of Horvath age acceleration among the three immobile social classes and the contribution of childhood social class to the Horvath age acceleration is higher than the contribution of adulthood social class (0.67 vs 0.33) when social mobility variable is not considered in the analysis (model 1, best model with smallest AIC or BIC). Horvath age acceleration is lower in women than men and there is a non-linear association with age (Supplementary Fig. S1). Results of DRM are similar when adding the mobility variables where no mobility variable is associated with Horvath indexed age acceleration.

**Table 3:** DRM parameters estimation [95% confidence interval] for age acceleration indexed by the Horvath algorithm, UK Household Longitudinal Study, 2010–2012.

	Model 1	Model 2	Model 3	Model 4
Disadvantage	0.16 [− 0.08, 0.41]	0.18 [− 0.08, 0.44]	0.21 [− 0.08, 0.51]	0.22 [− 0.05, 0.50]
Intermediate	− 0.11 [− 0.43, 0.22]	− 0.12 [− 0.38, 0.15]	− 0.11 [− 0.40, 0.19]	− 0.15 [− 0.45, 0.15]
Advantage	− 0.05 [− 0.41, 0.30]	− 0.06 [− 0.38, 0.25]	− 0.10 [− 0.50, 0.29]	− 0.08 [− 0.40, 0.25]
Origin occupation weight	0.67 [− 0.46, 1.81]	0.68 [− 0.18, 1.55]	0.44 [− 1.00, 1.87]	0.73 [− 0.42, 1.88]
Destination occupation weight	0.33 [− 0.81, 1.46]	0.32 [− 0.55, 1.18]	0.56 [− 0.87, 2.00]	0.27 [− 0.88, 1.42]
Female	− 0.96*** [− 1.22, − 0.70]	− 0.95*** [− 1.22, − 0.69]	− 0.95*** [− 1.21, − 0.69]	− 0.95*** [− 1.22, − 0.69]
Age	− 0.03 [− 0.16, 0.10]	− 0.03 [− 0.16, 0.10]	− 0.03 [− 0.16, 0.10]	− 0.03 [− 0.16, 0.10]
Age squared	− 0.35*** [− 0.47, − 0.24]	− 0.35*** [− 0.47, − 0.24]	− 0.35*** [− 0.47, − 0.24]	− 0.35*** [− 0.47, − 0.24]
Mobility in any direction		0.14 [− 0.13, 0.41]		
Downward mobility			0.03 [− 0.54, 0.61]	
Upward mobility			0.19 [− 0.23, 0.61]	
One-step downward mobility				0.10 [− 0.40, 0.61]
Two-step downward mobility				0.41 [− 0.82, 1.64]
One-step upward mobility				0.21 [− 0.11, 0.54]
Two-step upward mobility				− 0.04 [− 0.61, 0.52]
Constant	0.90*** [0.67, 1.13]	0.81*** [0.52, 1.10]	0.80*** [0.50, 1.10]	0.81*** [0.51, 1.10]
Observations	3140	3140	3140	3140
AIC	17,129.8	17,130.8	17,132.6	17,135.5
BIC	17,178.2	17,185.2	17,193.1	17,208.2

Models with covariates plus: (1) no mobility variable; (2) any direction mobility variable; (3) variables to indicate upward or downward trend; (4) variables to indicate one step, two step upward or downward trend. 95% confidence intervals in brackets.

***p < 0.001.

**Table 4:** DRM parameters estimation [95% confidence interval] for age acceleration indexed by Hannum algorithm, UK Household Longitudinal Study, 2010–2012.

	Model 1	Model 2	Model 3	Model 4
Disadvantage	0.27** [0.05, 0.49]	0.36*** [0.13, 0.58]	0.37*** [0.16, 0.59]	0.37*** [0.15, 0.60]
Intermediate	− 0.40*** [− 0.64, − 0.17]	− 0.13 [− 0.44, 0.19]	− 0.36** [− 0.62, − 0.10]	− 0.37** [− 0.64, − 0.10]
Advantage	0.14 [− 0.12, 0.39]	− 0.23 [− 0.51, 0.05]	− 0.02 [− 0.30, 0.27]	− 0.00 [− 0.29, 0.29]
Origin occupation weight	0.43** [0.12, 0.74]	1.00** [0.28, 1.73]	0.53** [0.17, 0.90]	0.53** [0.18, 0.88]
Destination occupation weight	0.57*** [0.26, 0.88]	− 0.00 [− 0.73, 0.72]	0.47* [0.10, 0.83]	0.47** [0.12, 0.82]
Female	− 1.73*** [− 1.96, − 1.51]	− 1.76*** [− 1.98, − 1.53]	− 1.74*** [− 1.96, − 1.51]	− 1.74*** [− 1.96, − 1.51]
Age	− 0.04 [− 0.15, 0.08]	− 0.06 [− 0.17, 0.05]	− 0.04 [− 0.16, 0.07]	− 0.04 [− 0.16, 0.07]
Age squared	− 0.23*** [− 0.32, − 0.13]	− 0.22*** [− 0.32, − 0.12]	− 0.22*** [− 0.32, − 0.13]	− 0.22*** [− 0.32, − 0.13]
Mobility in any direction		0.31* [0.04, 0.58]		
Downward mobility			0.18 [− 0.16, 0.53]	
Upward mobility			0.38** [0.12, 0.65]	
One-step downward mobility				0.20 [− 0.15, 0.55]
Two-step downward mobility				0.03 [− 0.88, 0.93]
One-step upward mobility				0.38** [0.10, 0.67]
Two-step upward mobility				0.37 [− 0.04, 0.79]
Constant	1.23*** [1.03, 1.43]	1.00*** [0.74, 1.25]	1.04*** [0.79, 1.29]	1.04*** [0.80, 1.29]
Observations	3105	3105	3105	3105
AIC	15,973.0	15,968.3	15,969.3	15,973.1
BIC	16,021.4	16,022.6	16,029.7	16,045.6

Models with covariates plus: (1) no mobility variable; (2) any direction mobility variable; (3) variables to indicate upward or downward trend; (4) variables to indicate one step, two step upward or downward trend.

95% confidence intervals in brackets.

*p < 0.05, **p < 0.01, ***p < 0.001.

**Table 5:** DRM parameters estimation [95% confidence interval] for age acceleration indexed by Phenoage algorithm.

	Model 1	Model 2	Model 3	Model 4
Disadvantage	1.02*** [0.70, 1.34]	1.06*** [0.74, 1.39]	1.01*** [0.64, 1.39]	1.05*** [0.69, 1.42]
Intermediate	− 0.54** [− 0.89, − 0.19]	− 0.53** [− 0.88, − 0.18]	− 0.54** [− 0.89, − 0.19]	− 0.65** [− 1.04, − 0.26]
Advantage	− 0.48* [− 0.85, − 0.10]	− 0.53** [− 0.91, − 0.15]	− 0.47* [− 0.91, − 0.04]	− 0.41 [− 0.84, 0.02]
Origin occupation weight	0.49*** [0.30, 0.69]	0.50*** [0.31, 0.68]	0.57** [0.23, 0.90]	0.73*** [0.38, 1.07]
Destination occupation weight	0.51*** [0.31, 0.70]	0.50*** [0.32, 0.69]	0.43* [0.10, 0.77]	0.27 [− 0.07, 0.62]
Female	− 0.10 [− 0.45, 0.24]	− 0.10 [− 0.44, 0.24]	− 0.10 [− 0.44, 0.24]	− 0.10 [− 0.45, 0.24]
Age	− 0.08 [− 0.25, 0.10]	− 0.08 [− 0.25, 0.10]	− 0.07 [− 0.25, 0.10]	− 0.08 [− 0.25, 0.10]
Age squared	− 0.41*** [− 0.56, − 0.27]	− 0.41*** [− 0.56, − 0.26]	− 0.41*** [− 0.56, − 0.26]	− 0.41*** [− 0.56, − 0.26]
Mobility in any direction		0.26 [− 0.10, 0.63]		
Downward mobility			0.42 [− 0.25, 1.08]	
Upward mobility			0.19 [− 0.27, 0.64]	
One-step downward mobility				0.65 [− 0.02, 1.32]
Two-step downward mobility				0.75 [− 0.88, 2.38]
One-step upward mobility				0.27 [− 0.17, 0.70]
Two-step upward mobility				− 0.47 [− 1.24, 0.30]
Constant	0.52*** [0.22, 0.82]	0.34 [− 0.04, 0.73]	0.36 [− 0.03, 0.74]	0.37 [− 0.01, 0.76]
Observations	3140	3140	3140	3140
AIC	18,851.2	18,851.2	18,852.9	18,853.4
BIC	18,899.6	18,905.6	18,913.4	18,926.0

Models with covariates plus: (1) no mobility variable; (2) any direction mobility variable; (3) variables to indicate upward or downward trend; (4) variables to indicate one step, two step upward or downward trend.

95% confidence intervals in brackets.

*p < 0.05, **p < 0.01, ***p < 0.001.

**Table 6:** DRM parameters estimation [95% confidence interval] for age acceleration indexed by DunedinPoAm algorithm.

	Model 1	Model 2	Model 3	Model 4
Disadvantage	1.02*** [0.78, 1.26]	1.04*** [0.80, 1.29]	1.09*** [0.83, 1.35]	1.10*** [0.83, 1.36]
Intermediate	− 0.15 [− 0.40, 0.11]	− 0.13 [− 0.38, 0.12]	− 0.12 [− 0.35, 0.11]	− 0.25 [− 0.54, 0.04]
Advantage	− 0.87*** [− 1.15, − 0.60]	− 0.91*** [− 1.19, − 0.64]	− 0.97*** [− 1.24, − 0.70]	− 0.85*** [− 1.16, − 0.54]
Origin occupation weight	0.36 [0.20, 0.52]	0.35 [0.20, 0.51]	0.17 [− 0.20, 0.53]	0.66 [0.04, 1.28]
Destination occupation weight	0.64*** [0.48, 0.80]	0.65*** [0.49, 0.80]	0.83*** [0.47, 1.20]	0.34 [− 0.28, 0.96]
Female	− 0.75*** [− 1.00, − 0.50]	− 0.75*** [− 0.99, − 0.50]	− 0.74*** [− 0.99, − 0.50]	− 0.74*** [− 0.99, − 0.49]
Age	− 0.11 [− 0.24, 0.01]	− 0.11 [− 0.24, 0.01]	− 0.11 [− 0.24, 0.01]	− 0.11 [− 0.24, 0.01]
Age squared	0.07 [− 0.04, 0.17]	0.07 [− 0.04, 0.18]	0.07 [− 0.04, 0.18]	0.07 [− 0.04, 0.18]
Mobility in any direction		0.19 [− 0.08, 0.46]		
Downward mobility			− 0.09 [− 0.68, 0.49]	
Upward mobility			0.46 [− 0.11, 1.03]	
One-step downward mobility				0.55 [− 0.29, 1.39]
Two-step downward mobility				0.44 [− 1.22, 2.10]
One-step upward mobility				0.04 [− 0.55, 0.63]
Two-step upward mobility				− 0.74 [− 1.99, 0.52]
Constant	0.38*** [0.16, 0.60]	0.26 [− 0.02, 0.54]	0.25 [− 0.03, 0.53]	0.27 [− 0.01, 0.56]
Observations	3140	3140	3140	3140
AIC	16,843.9	16,843.9	16,844.8	16,846.1
BIC	16,892.3	16,898.4	16,905.3	16,918.8

Notes: Models with covariates plus: (1) no mobility variable; (2) any direction mobility variable; (3) variables to indicate upward or downward trend; (4) variables to indicate one step, two step upward or downward trend.

95% confidence intervals in brackets.

***p < 0.001.

From Table 4, a positive age acceleration indexed with the Hannum algorithm is apparent in immobile socially disadvantage participants (0.27 [0.06, 0.49]) while immobile intermediate SEP has a negative value (− 0.41 [− 0.64, − 0.18]) indicating lifecourse disadvantage is associated with a positive age acceleration while lifecourse intermediate group younger epigenetic age than expected. The result of immobile socially advantage group is not significant. The contribution of childhood social class to Hannum age acceleration of mobile groups is lower than the contribution of adulthood social class (0.43 vs 0.57) when social mobility is not considered in the analysis (model 1). In these multivariate analyses, as observed with Horvath, there is negative age acceleration indexed by the Hannum algorithm in women and there is a nonlinear trend with age (Supplementary Fig. S2). Results of model 2 in Table 4 shows that mobility is associated with Hannum age acceleration (0.32 [0.04, 0.60]) and results of model 3 and model 4 further confirm that there is association of Hannum age acceleration with upward mobility (0.47 [0.19, 0.75]) and one step up mobility (0.47 [0.18, 0.76]).

Table 5 shows that life course disadvantage has a positive value (1.02 [0.70, 1.34]) in age acceleration indexed by Phenoage while immobile intermediate has a negative value (− 0.54 [− 0.89, − 0.19]) as does life course advantage (− 0.48 [− 0.85, − 0.10]). The childhood and adulthood social classes contribute almost equally to Phenoage age acceleration for mobile groups (0.49 vs 0.51 model 1). Phenoage age acceleration has the non-linear association with chronological age (Supplementary Fig. S3) apparent for the other measures of age acceleration but did not differ between men and women. For Phenoage age acceleration, the social mobile parameters are not significant (model 2-model 4 in Table 5).

From Table 6, a positive value (1.02 [0.78, 1.26]) in age acceleration indexed by DunedinPoAm is clear in socially disadvantage participants while life course advantage has a negative value (− 0.87 [− 1.15, − 0.60]). The contribution of childhood social class to DunedinPoAm AA of mobile groups is lower than the contribution of adulthood social class (0.36 vs 0.64) when social mobility is not considered in the analysis (model 1). For DunedinPoAm AA, the social mobile parameters are not significant (model 2-model 4 in Table 6).

### Sensitivity analyses

Tables S2–S5 in Supplementary File show that the association with age, sex, social classes, and mobility variables for age acceleration indexed by the four epigenetic measures of age acceleration was robust when we further control for marital status and highest education qualification. In these multivariate analyses, compared to participants who were married or cohabiting, all four measures of age acceleration were positive in divorced participants. No clear associations were identified between educational qualifications with age acceleration indexed by Horvath, Hannum or Phenoage while positive age acceleration was apparent in participants with lower educational attainment when indexed by DunedinPoAm.

**Figure 1:**
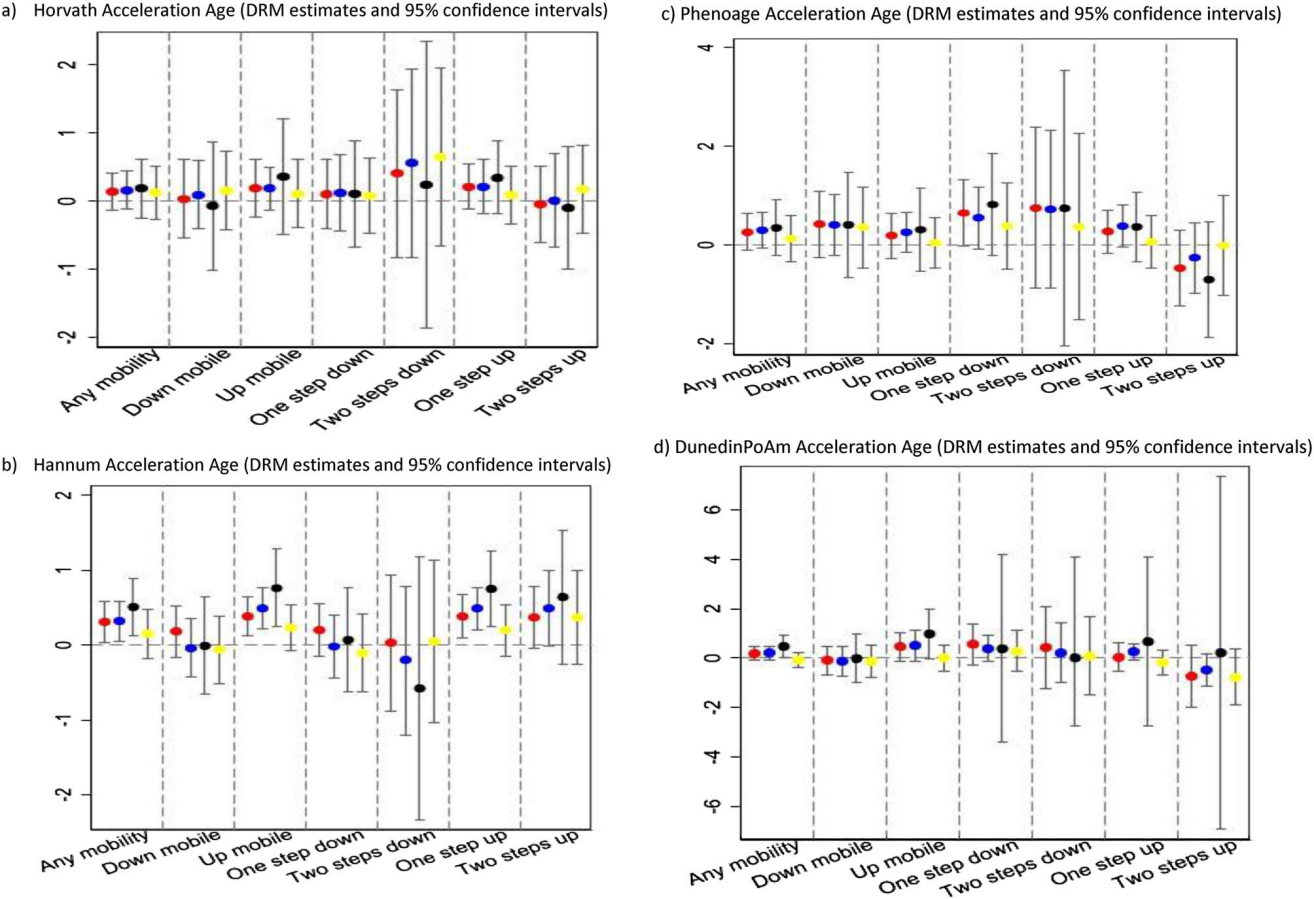
DRM estimate and 95% CI of mobility variables with (**a**) Horvath, (**b**) Hannum, (**c**) phenoage, and (**d**) DunedinPoAm Acceleration Age. Point estimates from the main model (red dot), adjusted for additional covariates, marital status and educational attainment (blue dot) and stratification of the sample to those born before 1956, the median year in the study (black dot) and those born after 1956 (yellow dot). Values above 0 represent positive accelerated age, i.e. older than chronological age and values below 0 represent negative accelerated age, i.e. younger than chronological age.

Tables S6–S9 in Supplementary Files showed that DRM estimates were robust when we define social class with individual social class rather than with the highest in the household. Tables S10–S17 in Supplementary Files describe the results based on birth year stratification (born before 1956 vs born after 1956) to investigate possible cohort effects on the findings. The upward mobility associations with Hannum were only confirmed in older participants (see Fig. 1). Figure 1 show that association of social mobility with age acceleration indexed by the four measures were robust to sensitivity analyses. As with Hannum and DunedinPoAm, the stably disadvantage group was identified older than expected in the older age group when measured with Phenoage but the association was not identified in the younger age group. Table 7 shows the association of mobile group with four algorithms restricting comparison groups to the two mobile-immobile comparisons. For downward vs immobile socially advantage comparison, there is a positive age acceleration indexed by DunedinPoAm only. For upward vs immobile socially disadvantage comparison, there is a negative age acceleration indexed by Phenoage and DunedinPoAm with the group moving upward from socially disadvantage origin.

**Table 7:** Ordinal linear regression estimates [95% confidence interval] for association of mobility vs immobility with accelerated age assessed with Horvath, Hannum, Phenoage and DunedinPoAm for two comparisons.

Model 1	Horvath	Hannum	Phenoage	DunedinPoAm
Downward mobile group (reference: immobile socially advantage group)	− 0.17 [− 0.97, 0.63]	− 0.35 [− 0.94, 0.25]	1.06 [− 0.03, 2.14]	0.96*** [0.24, 1.68]

Model 2	Horvath	Hannum	Phenoage	
Upward mobile group (reference: immobile socially disadvantage group)	0.13 [− 0.37, 0.63]	0.20 [− 0.21, 0.60]	− 0.65* [− 1.30, − 0.002]	− 0.96*** [− 1.47, − 0.46]

Model 1; downward mobile group vs immobile socially advantaged group.

Model 2; upward mobile group vs immobile socially disadvantage group. All models also controlled sex, standardized age and standardized age squared.

95% confidence intervals in brackets.

*p < 0.05, ***p < 0.001.

## Discussion

We find evidence for three lifecourse models, sensitive period, accumulation and social mobility in the association of SEP and age acceleration. Social position in early life is associated with age acceleration in adulthood. These observations were apparent for all estimations of age acceleration. Evidence for accumulation model was apparent for the second-generation algorithm. Phenoage indexed age acceleration with association observed for early life SEP and adult SEP and each stage contributing equally. Further, we find for this estimator, that upward mobility is associated with lower accelerated age compared to those who were disadvantaged throughout the lifecourse. We replicate our earlier observations of an association of early life SEP with the Hannum estimator of age acceleration^21^ and observe that this estimator was associated with upwards social position mobility across the lifecourse.

Our findings support the observations that early life social disadvantaged position is associated with accelerated age in later life^24,31–33^. We had shown an association of early life and age acceleration in later life, extend this to a wider and more representative subset of *Understanding Society*, responding to a previous critique that suggests that the current epigenetic literature is too focussed on convenience populations^34^.

This paper replicated an association of early life social position and a so called first generation measure of age acceleration and also describes associations of social position across the lifecourse with a second generation measure of age acceleration. The first generation measures were created to be predictive of age and are associated with mortality but less consistently with measures of social position. The second generation measures were created to be predictive of mortality and are more reflective of biological age^35^. For our first generation algorithm, our data do not accord with previous reports of the association of social mobility with age acceleration, that is we observe positive age acceleration association with upwards mobility, which is not in the same direction as previously reported^24,31,32^, where our data are statistically significant. This may be for a number of reasons: the analytic method we used which sought to separate mobility from origin and destination SEP^24,31,32^; the measure of social position used in the analyses^32^ and the measures of accelerated age used^32^. There is a wide body of literature on social mobility and broader measures of health; it is possible that the wider social mobility literature is mixed due to the failure to examine it independently of origin and destination SEP^36,37^. We^21^ and others^24^ have suggested that early life SEP and education attainment^23^ rather than later life are associated with age acceleration and other measures of health such as allostatic load^38^. These results add support to the evidence that early life has an impact on health throughout the lifecourse.

For the second generation measure we examined, when comparing mobility to those who were immobile for advantage and disadvantage throughout the lifecourse our observations with Phenoage and DunedinPoAm indexed accelerated age provide support for an accumulative lifecourse model and support the notion that social mobility serves to ameliorate health inequality.

Our suggestions of age acceleration indexed by Hannum with upward social mobility is novel but accords with other markers of health, such as inflammatory markers^39^. However, these patterns of association contrast to observations with other outcomes such as BMI^40^ and hypertension^41,42^ where associations with downward mobility rather than upward mobility are observed. Further we do not observe a dose response or stepwise association of increasing social position and age acceleration in our data suggesting that a move up one step is similar to a greater move up. This may be because there are few people that move more than one step and that most mobility is from intermediate groups. They also contrast with the restricted analyses and findings with age acceleration indexed by Phenoage and DunedinPoAm that suggest that upward mobility is associated with negative biological age acceleration compared to those that experience lifecourse disadvantage. These findings may further reflect the difference between the first and second generation algorithms.

Of the three models proposed to explain the link between life course SEP and adult disease^5^, our data support all three but they are specific to each index of age acceleration. Overall, these differences may reflect how each of the algorithms are generated, with the weakest associations apparent with the first-generation algorithm and stronger associations apparent with Phenoage and DunedinPoAm.

We suggest that our findings support the hypotheses that first generation estimates of accelerated age reflect processes until measurement and second generation estimates provide insights into processes after measurement^35^. However, our observation that early life disadvantage is associated with adult life age acceleration for the first generation measure to greater extent in older rather than younger cohorts requires replication. The observation can be explained in a number of ways, for example disadvantage in the childhoods of older people may be more adverse than childhood disadvantage experienced in younger people. Thus, conditions of disadvantage are likely to have been worse in absolute terms in childhoods in the 1930 and 1940s, which were characterised by depression and war than experienced in post 1950s when the UK was becoming a more equal country in the post war years^43^. A number of alternate explanations of the impact of upward mobility on adult health are suggested including the stress of cultural mismatch, physiological costs of striving and disruption of social connections^44^. We can speculate that mismatch between early and late life or ‘lack of belonging’ may have been more prevalent in older age groups than younger age groups as a greater proportion of the population enters higher education in the UK. In support, we observed the same pattern of association when we used education attainment rather than occupation in our analyses stratified by age (data not shown) suggesting the adverse association of social mobility may be ameliorated when higher education attainment is more normative. It is unclear how physiological processes^45^ and disruption of social connections, such as weakening connections with communities of origin and isolation^46^ and their association with age acceleration would vary by age group.

This study had several strengths as it is based on a national study, it comprised a large sample with representation from almost the entire adult age range. We examined a number of measures of age acceleration, using both first and second generation algorithms. We used a statistical method that explicitly examined the contribution of early (origin) and adult (destination) social position to age acceleration but it may generate results that underestimate mobility as it may implicitly force the mobility linear effect to zero^47^. However, when looking at early social position, we could not examine conditions in early childhood or in utero, where effects on DNAm age trajectories are plausibly stronger than at age 14 years. The sample was restricted to those reporting white/European ethnicity, meaning that results may not be generalizable to other ethnic groups. Measurement of DNAm was made on one occasion in adults and therefore it is not possible to examine whether age acceleration occurred before social mobility and further it is not possible to assess within person change in adulthood. We examined three models of social mobility and four age related outcomes, two of which were considered first generation. There are additional algorithms that are developed and can be tested. We did not adjust for multiple testing, as the strength of associations indicated that our main conclusions would not be altered by a Bonferroni correction. Many of associations with covariates have been reported previously, for example sex differences in accelerated age^48,49^ but others, such as the positive accelerated age we observe in divorced groups may requires further investigation. A focus on estimators of age restricts analyses to a very small subset of methylation across the genome and thus limits an analysis of SEP with DNA methylation more broadly. Broader analyses have the potential to uncover wider pathways by which SEP and health might be associated.

In conclusion we observe that disadvantaged social position in early life and adulthood is associated with positive age acceleration in adulthood. This is particularly apparent for adult SEP and the second generation measures of age acceleration. The social mobility framework is only partially supported with upwards social mobility associated with health measured with positive age acceleration.

## Supplementary Information

Supplementary Information.

## Data Availability

All data are available from the UKDA. University of Essex. Institute for Social and Economic Research and National Centre for Social Research, Understanding Society: Waves 2 and 3 Nurse Health Assessment, 2010–2012 [data collection]. 3rd Edition. UK Data Service. SN:7251. 10.5255/UKDA-SN-7251-3.
