# Supplementary material for: Social mobility across the lifecourse and DNA methylation age acceleration in adults in the UK
Source: Sci Rep. 2022 Dec 24;12:22284. doi: 10.1038/s41598-022-26433-2 (PMC9790005; doi:10.1038/s41598-022-26433-2)
Supplement: Supplementary file 1 — Supplementary Information. [file 41598_2022_26433_MOESM1_ESM.docx]

Social mobility across the lifecourse and DNA methylation age acceleration in adults in the UK

(Supplementary file)

Figure S1: the scatterplots of Horvath accelerated age vs standardized chronological age and the linear regression fitting line (in blue) and quadratic regression fitting curve (in green). Values above 0 represent positive age acceleration, values below 0 represent negative age acceleration.

Figure S2: the scatterplots of Hannum accelerated age vs standardized chronological age and the linear regression fitting line (in blue) and quadratic regression fitting curve (in green). Values above 0 represent positive age acceleration, values below 0 represent negative age acceleration.

Figure S3: the scatterplots of phenoage accelerated age vs standardized chronological age and the linear regression fitting line (in blue) and quadratic regression fitting curve (in green). Values above 0 represent positive age acceleration, values below 0 represent negative age acceleration.

Figure S4: the scatterplots of DunedinPoAm accelerated age vs standardized chronological age and the linear regression fitting line (in blue) and quadratic regression fitting curve (in green). Values above 0 represent positive age acceleration, values below 0 represent negative age acceleration.

Table S1: List of missing probes in algorithms algorithm with the Illumina Infinium HumanMethylationEIPIC array

| **Part 1: *Horvath 2013 Missing Probes (up to 20 probes)*** | | |
| --- | --- | --- |
| Probe ID | Chromosome | Gene Annotation |
| cg02654291 | 9 | C9orf64 |
| cg02972551 | 2 | KDM3A;KDM3A |
| cg09785172 | 4 | WFS1;WFS1;WFS1;WFS1 |
| cg09869858 | 12 | P11 |
| cg13682722 | 14 | C14orf102;C14orf102 |
| cg14329157 | 2 | WDR69 |
| cg16494477 | 5 | FGF18 |
| cg17408647 | 7 | C7orf44;C7orf44 |
| cg19167673^*^ | 22 | PDGFB |
| cg19273182 | 2 | PAPOLG;PAPOLG |
| cg19945840 | 1 | SDF4;SDF4;B3GALT6 |
| cg27319898 | 7 | ZNF804B;ZNF804B |
| cg27413543 | 4 | SEC31A |
| cg04431054 | 5 | PRRC1 |
| cg05590257 | 17 | PLD6 |
| cg06117855 | 3 | CLEC3B;CLEC3B |
| cg11025793 | 19 | IER2;STX10 |
| cg19046959 | 1 | COL8A2 |
| cg19569684 | 5 | MGC29506 |
| cg24471894 | 9 | KIAA0020 |
| cg27016307 | 19 | HRC |
| ^*^ Only missing for sample measured at 2020 (sample size 2,840) | | |
| **Part 2*: Hannum Missing Probes (7 probes)*** | | |
| Probe ID | Chromosome | Gene Annotation |
| cg24079702 | 2 | FHL2;FHL2;FHL2;FHL2 |
| cg14361627 | 7 | KLF14 |
| cg22285878 | 7 | KLF14 |
| cg07927379 | 7 | C7orf13;RNF32 |
| cg18473521 | 12 | HOXC4;HOXC4 |
| cg09651136 | 15 | PKM2;PKM2;PKM2 |
| cg21139312 | 17 | MSI2;MSI2 |
| **Part 3: PhenoAge *Missing Probes (up to 2 probes)*** | | |
| Probe ID | Chromosome | Gene Annotation |
| cg08212685 | 5 | ATG10 |
| cg26665419^**^ | 6 | C6orf203 |
| ^**^ Only missing for samples measured at 2017 (sample size 1,174) | | |

Table S2: DRM parameters estimates [95% confidence interval] for accelerated age indexed by Horvath, further controlled for marital status and highest educational qualification

|  | Model 1 | Model 2 | Model 3 | Model 4 |
| --- | --- | --- | --- | --- |
| Disadvantage | 0.12  [-0.15,0.40] | 0.13  [-0.18,0.43] | 0.14  [-0.22,0.50] | 0.16  [-0.14,0.46] |
| Intermediate | -0.05  [-0.26,0.17] | -0.08  [-0.31,0.14] | -0.09  [-0.34,0.15] | -0.09  [-0.39,0.21] |
| Advantage | -0.08  [-0.36,0.20] | -0.04  [-0.36,0.28] | -0.05  [-0.46,0.37] | -0.07  [-0.43,0.28] |
| Origin occupation weight | 1.07  [-0.76,2.89] | 0.97  [-0.63,2.56] | 0.73  [-1.16,2.61] | 1.00  [-1.38,3.37] |
| Destination occupation weight | -0.07  [-1.89,1.76] | 0.03  [-1.56,1.63] | 0.27  [-1.61,2.16] | 0.00  [-2.37,2.38] |
| Female | -1.03^***^  [-1.30,-0.77] | -1.03^***^  [-1.29,-0.77] | -1.03^***^  [-1.29,-0.77] | -1.03^***^  [-1.29,-0.77] |
| Age | -0.12  [-0.27,0.03] | -0.12  [-0.27,0.03] | -0.12  [-0.26,0.03] | -0.12  [-0.27,0.03] |
| Age squared | -0.34^***^  [-0.46,-0.22] | -0.34^***^  [-0.45,-0.22] | -0.34^***^  [-0.45,-0.22] | -0.34^***^  [-0.45,-0.22] |
| Single | -0.27  [-0.69,0.16] | -0.26  [-0.68,0.16] | -0.26  [-0.68,0.16] | -0.26  [-0.68,0.16] |
| Divorced | 0.51^**^  [0.18,0.84] | 0.51^**^  [0.18,0.84] | 0.51^**^  [0.18,0.84] | 0.51^**^  [0.18,0.84] |
| A-level | -0.25  [-0.62,0.12] | -0.25  [-0.62,0.12] | -0.24  [-0.62,0.13] | -0.24  [-0.62,0.13] |
| GCSE | -0.22  [-0.60,0.16] | -0.20  [-0.58,0.18] | -0.19  [-0.59,0.21] | -0.20  [-0.60,0.19] |
| No qualification | 0.04  [-0.49,0.57] | 0.07  [-0.46,0.61] | 0.08  [-0.47,0.64] | 0.07  [-0.47,0.61] |
| Mobility in any direction |  | 0.16  [-0.12,0.44] |  |  |
| Downward mobility |  |  | 0.09  [-0.41,0.60] |  |
| Upward mobility |  |  | 0.19  [-0.13,0.50] |  |
| One-step downward mobility |  |  |  | 0.12  [-0.44,0.68] |
| Two-step downward mobility |  |  |  | 0.56  [-0.82,1.94] |
| One-step upward mobility |  |  |  | 0.21  [-0.19,0.61] |
| Two-step upward mobility |  |  |  | 0.01  [-0.67,0.70] |
| Constant | 0.98^***^  [0.62,1.35] | 0.88^***^  [0.49,1.28] | 0.87^***^  [0.47,1.27] | 0.87^***^  [0.47,1.27] |
| Observations | 3,131 | 3,131 | 3,131 | 3,131 |
| AIC | 17055.1 | 17055.9 | 17057.8 | 17060.8 |
| BIC | 17133.8 | 17140.6 | 17148.5 | 17163.6 |

Notes: Models with covariates plus: 1) no mobility variable; 2) any direction mobility variable; 3) variables to indicate upward or downward trend; 4) variables to indicate one step, two step upward or downward trend95% confidence intervals in brackets. * p < .05, ** p < .01, *** p < .001.

Table S3: DRM parameters estimates [95% confidence interval] for accelerated age indexed by Hannum, further controlled for marital status and highest educational qualification

|  | Model 1 | Model 2 | Model 3 | Model 4 |
| --- | --- | --- | --- | --- |
| Disadvantage | 0.28^*^  [0.03,0.52] | 0.35^**^  [0.11,0.59] | 0.42^**^  [0.17,0.67] | 0.42^**^  [0.16,0.68] |
| Intermediate | -0.01  [-0.13,0.11] | -0.12  [-0.39,0.14] | -0.34^**^  [-0.57,-0.11] | -0.34^**^  [-0.60,-0.08] |
| Advantage | -0.26^*^  [-0.49,-0.04] | -0.22  [-0.48,0.03] | -0.08  [-0.37,0.21] | -0.08  [-0.38,0.23] |
| Origin occupation weight | 1.42  [0.57,2.26] | 1.04  [0.34,1.74] | 0.42  [0.07,0.77] | 0.42  [0.02,0.82] |
| Destination occupation weight | -0.42  [-1.26,0.43] | -0.04  [-0.74,0.66] | 0.58^**^  [0.23,0.93] | 0.58^**^  [0.18,0.98] |
| Female | -1.78^***^  [-2.01,-1.56] | -1.78^***^  [-2.00,-1.55] | -1.77^***^  [-1.99,-1.54] | -1.76^***^  [-1.99,-1.54] |
| Age | -0.09  [-0.22,0.04] | -0.09  [-0.21,0.04] | -0.08  [-0.21,0.05] | -0.08  [-0.21,0.05] |
| Age squared | -0.22^***^  [-0.32,-0.12] | -0.22^***^  [-0.32,-0.12] | -0.22^***^  [-0.32,-0.12] | -0.22^***^  [-0.32,-0.12] |
| Single | -0.02  [-0.38,0.34] | -0.03  [-0.39,0.33] | -0.03  [-0.39,0.33] | -0.03  [-0.39,0.33] |
| Divorced | 0.30^*^  [0.02,0.58] | 0.28  [-0.00,0.56] | 0.29^*^  [0.01,0.57] | 0.29^*^  [0.01,0.57] |
| A-level | -0.24  [-0.56,0.08] | -0.26  [-0.58,0.05] | -0.19  [-0.51,0.13] | -0.19  [-0.52,0.13] |
| GCSE | -0.13  [-0.47,0.21] | -0.15  [-0.48,0.17] | -0.06  [-0.40,0.28] | -0.06  [-0.40,0.28] |
| No qualification | -0.06  [-0.53,0.42] | -0.09  [-0.55,0.37] | -0.03  [-0.49,0.44] | -0.03  [-0.49,0.44] |
| Mobility in any direction |  | 0.32^*^  [0.05,0.59] |  |  |
| Downward mobility |  |  | -0.04  [-0.43,0.36] |  |
| Upward mobility |  |  | 0.49^***^  [0.21,0.77] |  |
| One-step downward mobility |  |  |  | -0.02  [-0.44,0.40] |
| Two-step downward mobility |  |  |  | -0.20  [-1.20,0.79] |
| One-step upward mobility |  |  |  | 0.49^***^  [0.20,0.77] |
| Two-step upward mobility |  |  |  | 0.49  [-0.01,1.00] |
| Constant | 1.19^***^  [0.85,1.52] | 1.08^***^  [0.73,1.43] | 1.03^***^  [0.69,1.38] | 1.04^***^  [0.69,1.38] |
| Observations | 3,131 | 3,131 | 3,131 | 3,131 |
| AIC | 16087.2 | 16084.0 | 16080.4 | 16084.2 |
| BIC | 16165.8 | 16168.7 | 16171.1 | 16187.1 |

Notes: Models with covariates plus: 1) no mobility variable; 2) any direction mobility variable; 3) variables to indicate upward or downward trend; 4) variables to indicate one step, two step upward or downward trend. 95% confidence intervals in brackets. * p < .05, ** p < .01, *** p < .001.

Table S4: DRM parameters estimates [95% confidence interval] for accelerated age indexed by Phenoage, further controlled for marital status and highest educational qualification

|  | Model 1 | Model 2 | Model 3 | Model 4 |
| --- | --- | --- | --- | --- |
| Disadvantage | 0.75^***^  [0.38,1.12] | 0.78^***^  [0.40,1.16] | 0.75^***^  [0.34,1.16] | 0.81^***^  [0.41,1.20] |
| Intermediate | -0.52^**^  [-0.88,-0.16] | -0.52^**^  [-0.86,-0.17] | -0.52^**^  [-0.86,-0.17] | -0.61^**^  [-1.01,-0.21] |
| Advantage | -0.23  [-0.67,0.20] | -0.26  [-0.71,0.18] | -0.23  [-0.70,0.24] | -0.20  [-0.66,0.27] |
| Origin occupation weight | 0.57  [0.32,0.83] | 0.59  [0.34,0.84] | 0.65  [0.26,1.03] | 0.76  [0.39,1.13] |
| Destination occupation weight | 0.43^**^  [0.17,0.68] | 0.41^**^  [0.16,0.66] | 0.35  [-0.03,0.74] | 0.24  [-0.13,0.61] |
| Female | -0.26  [-0.61,0.08] | -0.26  [-0.60,0.09] | -0.26  [-0.61,0.08] | -0.26  [-0.61,0.08] |
| Age | -0.19  [-0.38,0.00] | -0.19  [-0.39,0.00] | -0.19  [-0.39,0.00] | -0.19  [-0.39,0.00] |
| Age squared | -0.44^***^  [-0.59,-0.29] | -0.44^***^  [-0.59,-0.29] | -0.44^***^  [-0.59,-0.29] | -0.44^***^  [-0.59,-0.29] |
| Single | 0.33  [-0.22,0.88] | 0.34  [-0.21,0.89] | 0.34  [-0.22,0.89] | 0.33  [-0.22,0.89] |
| Divorced | 1.21^***^  [0.78,1.64] | 1.22^***^  [0.79,1.65] | 1.21^***^  [0.78,1.64] | 1.21^***^  [0.78,1.64] |
| A-level | 0.21  [-0.29,0.70] | 0.20  [-0.29,0.69] | 0.19  [-0.30,0.68] | 0.21  [-0.28,0.70] |
| GCSE | 0.21  [-0.30,0.73] | 0.23  [-0.29,0.75] | 0.22  [-0.30,0.74] | 0.23  [-0.29,0.75] |
| No qualification | 0.71^*^  [0.00,1.42] | 0.76^*^  [0.04,1.47] | 0.76^*^  [0.04,1.47] | 0.75^*^  [0.04,1.45] |
| Mobility in any direction |  | 0.30  [-0.06,0.67] |  |  |
| Downward mobility |  |  | 0.41  [-0.21,1.03] |  |
| Upward mobility |  |  | 0.26  [-0.15,0.67] |  |
| One-step downward mobility |  |  |  | 0.55  [-0.08,1.18] |
| Two-step downward mobility |  |  |  | 0.72  [-0.88,2.32] |
| One-step upward mobility |  |  |  | 0.38  [-0.05,0.81] |
| Two-step upward mobility |  |  |  | -0.26  [-0.97,0.45] |
| Constant | 0.10  [-0.35,0.56] | -0.10  [-0.62,0.42] | -0.09  [-0.62,0.43] | -0.09  [-0.61,0.44] |
| Observations | 3,131 | 3,131 | 3,131 | 3,131 |
| AIC | 18759.9 | 18759.2 | 18761.0 | 18762.0 |
| BIC | 18838.6 | 18843.9 | 18851.8 | 18864.8 |

Models with covariates plus: 1) no mobility variable; 2) any direction mobility variable; 3) variables to indicate upward or downward trend; 4) variables to indicate one step, two step upward or downward trend. Notes: 95% confidence intervals in brackets. * p < .05, ** p < .01, *** p < .001.

Table S5: DRM parameters estimates [95% confidence interval] for accelerated age indexed by DunedinPoAm, further controlled for marital status and highest educational qualification

|  | Model 1 | Model 2 | Model 3 | Model 4 |
| --- | --- | --- | --- | --- |
| Disadvantage | 0.56^***^  [0.30,0.82] | 0.58^***^  [0.32,0.85] | 0.64^***^  [0.35,0.93] | 0.65^***^  [0.37,0.93] |
| Intermediate | -0.19  [-0.45,0.06] | -0.17  [-0.42,0.08] | -0.11  [-0.35,0.13] | -0.31^*^  [-0.60,-0.03] |
| Advantage | -0.37^*^  [-0.65,-0.08] | -0.42^**^  [-0.70,-0.13] | -0.53^***^  [-0.81,-0.24] | -0.34  [-0.67,0.00] |
| Origin occupation weight | 0.36  [0.08,0.64] | 0.36  [0.09,0.62] | 0.01  [-0.67,0.68] | 0.63  [0.15,1.12] |
| Destination occupation weight | 0.64^***^  [0.36,0.92] | 0.64^***^  [0.38,0.91] | 0.99^**^  [0.32,1.67] | 0.37  [-0.12,0.85] |
| Female | -0.91^***^  [-1.16,-0.66] | -0.91^***^  [-1.16,-0.66] | -0.91^***^  [-1.16,-0.66] | -0.91^***^  [-1.16,-0.66] |
| Age | -0.26^***^  [-0.40,-0.12] | -0.26^***^  [-0.40,-0.12] | -0.26^***^  [-0.40,-0.12] | -0.26^***^  [-0.40,-0.12] |
| Age squared | 0.03  [-0.08,0.14] | 0.03  [-0.08,0.14] | 0.03  [-0.08,0.14] | 0.03  [-0.08,0.14] |
| Single | 0.44^*^  [0.04,0.84] | 0.44^*^  [0.04,0.84] | 0.45^*^  [0.05,0.85] | 0.45^*^  [0.05,0.85] |
| Divorced | 1.03^***^  [0.72,1.34] | 1.03^***^  [0.72,1.34] | 1.03^***^  [0.72,1.35] | 1.03^***^  [0.72,1.34] |
| A-level | 0.68^***^  [0.33,1.04] | 0.67^***^  [0.31,1.02] | 0.67^***^  [0.32,1.02] | 0.70^***^  [0.35,1.06] |
| GCSE | 1.04^***^  [0.67,1.42] | 1.04^***^  [0.66,1.41] | 1.03^***^  [0.66,1.40] | 1.08^***^  [0.70,1.45] |
| No qualification | 1.66^***^  [1.15,2.18] | 1.68^***^  [1.16,2.19] | 1.67^***^  [1.16,2.18] | 1.69^***^  [1.18,2.20] |
| Mobility in any direction |  | 0.21  [-0.06,0.47] |  |  |
| Downward mobility |  |  | -0.13  [-0.73,0.47] |  |
| Upward mobility |  |  | 0.50  [-0.11,1.11] |  |
| One-step downward mobility |  |  |  | 0.39  [-0.15,0.92] |
| Two-step downward mobility |  |  |  | 0.20  [-1.00,1.40] |
| One-step upward mobility |  |  |  | 0.25  [-0.08,0.59] |
| Two-step upward mobility |  |  |  | -0.48  [-1.12,0.17] |
| Constant | -0.57^**^  [-0.90,-0.23] | -0.69^***^  [-1.07,-0.32] | -0.71^***^  [-1.09,-0.33] | -0.70^***^  [-1.08,-0.32] |
| Observations | 3,131 | 3,131 | 3,131 | 3,131 |
| AIC | 16715.8 | 16715.5 | 16716.0 | 16715.8 |
| BIC | 16794.4 | 16800.2 | 16806.7 | 16818.7 |

Models with covariates plus: 1) no mobility variable; 2) any direction mobility variable; 3) variables to indicate upward or downward trend; 4) variables to indicate one step, two step upward or downward trend. Notes: 95% confidence intervals in brackets. * p < .05, ** p < .01, *** p < .001.

Table S6: DRM parameters estimates [95% confidence interval] for accelerated age indexed by Horvath, where childhood social class is mainly defined by father’s occupation (or mother’s occupation when father’s missing) and adulthood social class is defined by own occupation

|  | Model 1 | Model 2 | Model 3 | Model 4 |
| --- | --- | --- | --- | --- |
| Disadvantage | 0.08  [-0.15,0.30] | 0.09  [-0.15,0.33] | 0.16  [-0.11,0.42] | 0.15  [-0.11,0.42] |
| Intermediate | -0.12  [-0.37,0.13] | -0.12  [-0.37,0.13] | -0.09  [-0.33,0.15] | -0.11  [-0.41,0.20] |
| Advantage | 0.05  [-0.16,0.25] | 0.03  [-0.22,0.28] | -0.07  [-0.35,0.21] | -0.05  [-0.39,0.30] |
| Origin occupation weight | 0.15  [-1.06,1.35] | 0.24  [-0.94,1.42] | -0.16  [-2.08,1.77] | 0.02  [-2.04,2.07] |
| Destination occupation weight | 0.85  [-0.35,2.06] | 0.76  [-0.42,1.94] | 1.16  [-0.77,3.08] | 0.98  [-1.07,3.04] |
| Female | -0.94^***^  [-1.20,-0.67] | -0.94^***^  [-1.20,-0.68] | -0.94^***^  [-1.20,-0.68] | -0.94^***^  [-1.20,-0.68] |
| Age | -0.03  [-0.16,0.10] | -0.03  [-0.16,0.10] | -0.03  [-0.17,0.10] | -0.03  [-0.17,0.10] |
| Age squared | -0.37^***^  [-0.48,-0.25] | -0.36^***^  [-0.48,-0.25] | -0.36^***^  [-0.48,-0.25] | -0.36^***^  [-0.48,-0.25] |
| Mobility in any direction |  | 0.07  [-0.24,0.37] |  |  |
| Downward mobility |  |  | -0.11  [-0.65,0.43] |  |
| Upward mobility |  |  | 0.20  [-0.31,0.72] |  |
| One-step downward mobility |  |  |  | -0.08  [-0.66,0.50] |
| Two-step downward mobility |  |  |  | 0.01  [-1.09,1.11] |
| One-step upward mobility |  |  |  | 0.18  [-0.26,0.63] |
| Two-step upward mobility |  |  |  | 0.12  [-0.75,0.99] |
| Constant | 0.88^***^  [0.64,1.11] | 0.84^***^  [0.55,1.13] | 0.81^***^  [0.52,1.10] | 0.82^***^  [0.52,1.11] |
| Observations | 3,105 | 3,105 | 3,105 | 3,105 |
| AIC | 16930.1 | 16931.9 | 16933.1 | 16937.1 |
| BIC | 16978.4 | 16986.3 | 16993.5 | 17009.6 |

Models with covariates plus: 1) no mobility variable; 2) any direction mobility variable; 3) variables to indicate upward or downward trend; 4) variables to indicate one step, two step upward or downward trend. Notes: 95% confidence intervals in brackets. * p < .05, ** p < .01, *** p < .001.

Table S7: DRM parameters estimates [95% confidence interval] for accelerated age indexed by Hannum, where childhood social class is mainly defined by father’s occupation (or mother’s occupation when father’s missing) and adulthood social class is defined by own occupation

|  | Model 1 | Model 2 | Model 3 | Model 4 |
| --- | --- | --- | --- | --- |
| Disadvantage | 0.25^*^  [0.04,0.45] | 0.32^**^  [0.11,0.53] | 0.40^***^  [0.18,0.63] | 0.40^***^  [0.18,0.63] |
| Intermediate | -0.37^***^  [-0.59,-0.15] | -0.33^**^  [-0.58,-0.08] | -0.31^**^  [-0.53,-0.10] | -0.29^*^  [-0.55,-0.03] |
| Advantage | 0.13  [-0.09,0.34] | 0.01  [-0.28,0.31] | -0.09  [-0.35,0.17] | -0.12  [-0.41,0.18] |
| Origin occupation weight | 0.32  [0.01,0.64] | 0.47  [0.11,0.83] | 0.26  [-0.13,0.64] | 0.19  [-0.37,0.75] |
| Destination occupation weight | 0.68^***^  [0.36,0.99] | 0.53^**^  [0.17,0.89] | 0.74^***^  [0.36,1.13] | 0.81^**^  [0.25,1.37] |
| Female | -1.74^***^  [-1.96,-1.51] | -1.74^***^  [-1.97,-1.52] | -1.74^***^  [-1.96,-1.51] | -1.74^***^  [-1.96,-1.51] |
| Age | -0.04  [-0.15,0.08] | -0.04  [-0.15,0.07] | -0.04  [-0.16,0.07] | -0.04  [-0.16,0.07] |
| Age squared | -0.22^***^  [-0.32,-0.13] | -0.22^***^  [-0.32,-0.13] | -0.22^***^  [-0.32,-0.12] | -0.22^***^  [-0.32,-0.13] |
| Mobility in any direction |  | 0.28^*^  [0.03,0.53] |  |  |
| Downward mobility |  |  | -0.00  [-0.39,0.39] |  |
| Upward mobility |  |  | 0.44^**^  [0.12,0.76] |  |
| One-step downward mobility |  |  |  | -0.03  [-0.49,0.43] |
| Two-step downward mobility |  |  |  | -0.12  [-1.03,0.80] |
| One-step upward mobility |  |  |  | 0.43^*^  [0.09,0.77] |
| Two-step upward mobility |  |  |  | 0.56  [-0.09,1.20] |
| Constant | 1.19^***^  [0.99,1.39] | 1.03^***^  [0.78,1.28] | 0.99^***^  [0.74,1.24] | 0.99^***^  [0.73,1.24] |
| Observations | 3,105 | 3,105 | 3,105 | 3,105 |
| AIC | 15973.5 | 15970.9 | 15969.0 | 15972.8 |
| BIC | 16021.8 | 16025.2 | 16029.4 | 16045.2 |

Models with covariates plus: 1) no mobility variable; 2) any direction mobility variable; 3) variables to indicate upward or downward trend; 4) variables to indicate one step, two step upward or downward trend. Notes: 95% confidence intervals in brackets. * p < .05, ** p < .01, *** p < .001.

Table S8: DRM parameters estimates [95% confidence interval] for accelerated age indexed by Phenoage, where childhood social class is mainly defined by father’s occupation (or mother’s occupation when father’s missing) and adulthood social class is defined by own occupation

|  | Model 1 | Model 2 | Model 3 | Model 4 |
| --- | --- | --- | --- | --- |
| Disadvantage | 0.85^***^  [0.54,1.16] | 0.88^***^  [0.56,1.20] | 0.94^***^  [0.58,1.29] | 0.94^***^  [0.59,1.29] |
| Intermediate | -0.41^*^  [-0.76,-0.06] | -0.41^*^  [-0.76,-0.06] | -0.36  [-0.76,0.03] | -0.59^**^  [-0.99,-0.19] |
| Advantage | -0.44^*^  [-0.83,-0.05] | -0.47^*^  [-0.87,-0.08] | -0.57^*^  [-1.07,-0.07] | -0.35  [-0.80,0.10] |
| Origin occupation weight | 0.51  [0.28,0.73] | 0.51  [0.29,0.72] | 0.37  [-0.13,0.87] | 0.69  [0.33,1.05] |
| Destination occupation weight | 0.49^***^  [0.27,0.72] | 0.49^***^  [0.28,0.71] | 0.63^*^  [0.13,1.13] | 0.31  [-0.05,0.67] |
| Female | -0.09  [-0.44,0.25] | -0.09  [-0.44,0.25] | -0.09  [-0.44,0.25] | -0.10  [-0.45,0.25] |
| Age | -0.07  [-0.25,0.10] | -0.07  [-0.25,0.10] | -0.07  [-0.25,0.10] | -0.08  [-0.25,0.10] |
| Age squared | -0.42^***^  [-0.56,-0.27] | -0.41^***^  [-0.56,-0.27] | -0.42^***^  [-0.56,-0.27] | -0.41^***^  [-0.56,-0.26] |
| Mobility in any direction |  | 0.16  [-0.21,0.52] |  |  |
| Downward mobility |  |  | -0.05  [-0.84,0.73] |  |
| Upward mobility |  |  | 0.32  [-0.36,1.00] |  |
| One-step downward mobility |  |  |  | 0.38  [-0.27,1.03] |
| Two-step downward mobility |  |  |  | 0.31  [-1.18,1.79] |
| One-step upward mobility |  |  |  | 0.31  [-0.14,0.77] |
| Two-step upward mobility |  |  |  | -0.67  [-1.42,0.08] |
| Constant | 0.41^**^  [0.11,0.71] | 0.31  [-0.07,0.69] | 0.28  [-0.11,0.67] | 0.34  [-0.05,0.72] |
| Observations | 3,105 | 3,105 | 3,105 | 3,105 |
| AIC | 18645.8 | 18647.1 | 18648.7 | 18646.7 |
| BIC | 18694.2 | 18701.5 | 18709.1 | 18719.2 |

Models with covariates plus: 1) no mobility variable; 2) any direction mobility variable; 3) variables to indicate upward or downward trend; 4) variables to indicate one step, two step upward or downward trend. Notes: 95% confidence intervals in brackets. * p < .05, ** p < .01, *** p < .001.

Table S9: DRM parameters estimates [95% confidence interval] for accelerated age indexed by DunedinPoAm, where childhood social class is mainly defined by father’s occupation (or mother’s occupation when father’s missing) and adulthood social class is defined by own occupation

|  | Model 1 | Model 2 | Model 3 | Model 4 |
| --- | --- | --- | --- | --- |
| Disadvantage | 0.97^***^  [0.74,1.20] | 0.97^***^  [0.74,1.21] | 1.04^***^  [0.79,1.29] | 1.04^***^  [0.79,1.29] |
| Intermediate | -0.12  [-0.37,0.14] | -0.11  [-0.37,0.14] | -0.12  [-0.33,0.09] | -0.20  [-0.49,0.09] |
| Advantage | -0.85^***^  [-1.14,-0.57] | -0.86^***^  [-1.15,-0.57] | -0.92^***^  [-1.18,-0.66] | -0.84^***^  [-1.17,-0.51] |
| Origin occupation weight | 0.37  [0.21,0.54] | 0.37  [0.21,0.54] | 0.09  [-0.29,0.46] | 0.42  [-0.33,1.17] |
| Destination occupation weight | 0.63^***^  [0.46,0.79] | 0.63^***^  [0.46,0.79] | 0.91^***^  [0.54,1.29] | 0.58  [-0.17,1.33] |
| Female | -0.76^***^  [-1.01,-0.51] | -0.76^***^  [-1.01,-0.51] | -0.75^***^  [-1.00,-0.50] | -0.76^***^  [-1.01,-0.51] |
| Age | -0.10  [-0.22,0.03] | -0.10  [-0.22,0.03] | -0.10  [-0.22,0.03] | -0.10  [-0.22,0.03] |
| Age squared | 0.08  [-0.03,0.19] | 0.08  [-0.03,0.19] | 0.08  [-0.03,0.19] | 0.08  [-0.03,0.19] |
| Mobility in any direction |  | 0.01  [-0.25,0.28] |  |  |
| Downward mobility |  |  | -0.40  [-0.97,0.17] |  |
| Upward mobility |  |  | 0.42  [-0.16,0.99] |  |
| One-step downward mobility |  |  |  | 0.01  [-0.94,0.97] |
| Two-step downward mobility |  |  |  | -0.05  [-1.74,1.64] |
| One-step upward mobility |  |  |  | 0.12  [-0.60,0.84] |
| Two-step upward mobility |  |  |  | -0.37  [-1.93,1.19] |
| Constant | 0.27^*^  [0.05,0.49] | 0.26  [-0.02,0.54] | 0.24  [-0.04,0.52] | 0.26  [-0.02,0.54] |
| Observations | 3,105 | 3,105 | 3,105 | 3,105 |
| AIC | 16643.4 | 16645.4 | 16644.8 | 16647.9 |
| BIC | 16691.7 | 16699.8 | 16705.2 | 16720.4 |

Models with covariates plus: 1) no mobility variable; 2) any direction mobility variable; 3) variables to indicate upward or downward trend; 4) variables to indicate one step, two step upward or downward trend. Notes: 95% confidence intervals in brackets. * p < .05, ** p < .01, *** p < .001.

Table S10: DRM parameters estimates [95% confidence interval] for accelerated age indexed by Horvath for participants born before 1956

|  | Model 1 | Model 2 | Model 3 | Model 4 |
| --- | --- | --- | --- | --- |
| Disadvantage | 0.34  [-0.05,0.72] | 0.37  [-0.03,0.77] | 0.45^*^  [0.00,0.90] | 0.44^*^  [0.01,0.87] |
| Intermediate | -0.19  [-0.70,0.32] | -0.20  [-0.64,0.23] | -0.18  [-0.66,0.30] | -0.28  [-0.77,0.20] |
| Advantage | -0.15  [-0.76,0.46] | -0.17  [-0.73,0.39] | -0.27  [-0.93,0.39] | -0.16  [-0.74,0.43] |
| Origin occupation weight | 0.75  [-0.12,1.63] | 0.73  [0.04,1.41] | 0.38  [-0.88,1.64] | 0.69  [-0.20,1.57] |
| Destination occupation weight | 0.25  [-0.63,1.12] | 0.27  [-0.41,0.96] | 0.62  [-0.64,1.88] | 0.31  [-0.57,1.20] |
| Female | -0.97^***^  [-1.38,-0.55] | -0.96^***^  [-1.37,-0.55] | -0.96^***^  [-1.37,-0.55] | -0.96^***^  [-1.37,-0.55] |
| Age | 0.06  [-0.18,0.29] | 0.06  [-0.18,0.29] | 0.05  [-0.18,0.29] | 0.06  [-0.18,0.29] |
| Age squared | -0.10  [-0.25,0.05] | -0.10  [-0.25,0.05] | -0.10  [-0.25,0.05] | -0.10  [-0.25,0.05] |
| Mobility in any direction |  | 0.19  [-0.25,0.62] |  |  |
| Downward mobility |  |  | -0.07  [-1.02,0.87] |  |
| Upward mobility |  |  | 0.36  [-0.49,1.21] |  |
| One-step downward mobility |  |  |  | 0.11  [-0.67,0.89] |
| Two-step downward mobility |  |  |  | 0.24  [-1.86,2.34] |
| One-step upward mobility |  |  |  | 0.34  [-0.19,0.88] |
| Two-step upward mobility |  |  |  | -0.10  [-0.99,0.80] |
| Constant | 0.61^**^  [0.24,0.98] | 0.49^*^  [0.03,0.94] | 0.45  [-0.01,0.92] | 0.49^*^  [0.03,0.95] |
| Observations | 1,522 | 1,522 | 1,522 | 1,522 |
| AIC | 8606.6 | 8607.9 | 8609.4 | 8612.5 |
| BIC | 8649.2 | 8655.8 | 8662.7 | 8676.4 |

Models with covariates plus: 1) no mobility variable; 2) any direction mobility variable; 3) variables to indicate upward or downward trend; 4) variables to indicate one step, two step upward or downward trend. Notes: 95% confidence intervals in brackets. * p < .05, ** p < .01, *** p < .001.

Table S11: DRM parameters estimates [95% confidence interval] for accelerated age indexed by Hannum for participants born before 1956

|  | Model 1 | Model 2 | Model 3 | Model 4 |
| --- | --- | --- | --- | --- |
| Disadvantage | 0.51^**^  [0.17,0.84] | 0.61^***^  [0.27,0.94] | 0.72^***^  [0.35,1.09] | 0.72^***^  [0.34,1.09] |
| Intermediate | -0.04  [-0.31,0.23] | -0.33  [-1.00,0.34] | -0.46^*^  [-0.83,-0.10] | -0.51^*^  [-0.93,-0.09] |
| Advantage | -0.47^*^  [-0.86,-0.08] | -0.28  [-1.01,0.46] | -0.26  [-0.72,0.21] | -0.21  [-0.71,0.30] |
| Origin occupation weight | 1.15  [0.61,1.70] | 0.77  [0.13,1.40] | 0.38  [-0.02,0.78] | 0.42  [-0.02,0.85] |
| Destination occupation weight | -0.15  [-0.70,0.39] | 0.23  [-0.40,0.87] | 0.62^**^  [0.22,1.02] | 0.58^**^  [0.15,1.02] |
| Female | -1.89^***^  [-2.25,-1.53] | -1.88^***^  [-2.24,-1.52] | -1.86^***^  [-2.22,-1.50] | -1.85^***^  [-2.21,-1.49] |
| Age | 0.00  [-0.20,0.21] | -0.00  [-0.20,0.20] | 0.00  [-0.20,0.21] | 0.01  [-0.20,0.21] |
| Age squared | -0.04  [-0.17,0.10] | -0.03  [-0.17,0.10] | -0.03  [-0.17,0.10] | -0.04  [-0.17,0.10] |
| Mobility in any direction |  | 0.51^**^  [0.13,0.89] |  |  |
| Downward mobility |  |  | -0.01  [-0.66,0.64] |  |
| Upward mobility |  |  | 0.76^**^  [0.24,1.28] |  |
| One-step downward mobility |  |  |  | 0.07  [-0.62,0.76] |
| Two-step downward mobility |  |  |  | -0.58  [-2.34,1.18] |
| One-step upward mobility |  |  |  | 0.75^**^  [0.25,1.25] |
| Two-step upward mobility |  |  |  | 0.64  [-0.26,1.53] |
| Constant | 0.89^***^  [0.55,1.24] | 0.70^**^  [0.27,1.13] | 0.69^***^  [0.29,1.09] | 0.70^***^  [0.30,1.11] |
| Observations | 1,522 | 1,522 | 1,522 | 1,522 |
| AIC | 8200.9 | 8197.1 | 8195.2 | 8198.7 |
| BIC | 8243.5 | 8245.0 | 8248.5 | 8262.6 |

Models with covariates plus: 1) no mobility variable; 2) any direction mobility variable; 3) variables to indicate upward or downward trend; 4) variables to indicate one step, two step upward or downward trend. Notes: 95% confidence intervals in brackets. * p < .05, ** p < .01, *** p < .001.

Table S12: DRM parameters estimates [95% confidence interval] for accelerated age indexed by Phenoage for participants born before 1956

|  | Model 1 | Model 2 | Model 3 | Model 4 |
| --- | --- | --- | --- | --- |
| Disadvantage | 1.13^***^  [0.65,1.61] | 1.21^***^  [0.72,1.70] | 1.19^***^  [0.61,1.77] | 1.20^***^  [0.66,1.74] |
| Intermediate | -0.53  [-1.07,0.01] | -0.51  [-1.04,0.02] | -0.52  [-1.07,0.03] | -0.69^*^  [-1.29,-0.08] |
| Advantage | -0.60  [-1.24,0.05] | -0.70^*^  [-1.36,-0.04] | -0.67  [-1.46,0.12] | -0.51  [-1.25,0.22] |
| Origin occupation weight | 0.58  [0.32,0.84] | 0.57  [0.32,0.81] | 0.60  [0.08,1.12] | 0.83  [0.32,1.34] |
| Destination occupation weight | 0.42^**^  [0.16,0.68] | 0.43^***^  [0.19,0.68] | 0.40  [-0.12,0.92] | 0.17  [-0.34,0.68] |
| Female | -0.51  [-1.03,0.01] | -0.51  [-1.03,0.01] | -0.51  [-1.03,0.01] | -0.51  [-1.03,0.01] |
| Age | -0.05  [-0.34,0.25] | -0.05  [-0.35,0.24] | -0.05  [-0.35,0.24] | -0.05  [-0.35,0.24] |
| Age squared | 0.00  [-0.19,0.19] | 0.00  [-0.19,0.19] | 0.00  [-0.19,0.19] | -0.00  [-0.19,0.19] |
| Mobility in any direction |  | 0.35  [-0.21,0.91] |  |  |
| Downward mobility |  |  | 0.41  [-0.66,1.48] |  |
| Upward mobility |  |  | 0.31  [-0.54,1.15] |  |
| One-step downward mobility |  |  |  | 0.82  [-0.21,1.85] |
| Two-step downward mobility |  |  |  | 0.74  [-2.05,3.53] |
| One-step upward mobility |  |  |  | 0.37  [-0.34,1.07] |
| Two-step upward mobility |  |  |  | -0.70  [-1.88,0.47] |
| Constant | 0.23  [-0.20,0.66] | -0.01  [-0.58,0.56] | 0.00  [-0.59,0.59] | 0.06  [-0.53,0.64] |
| Observations | 1,522 | 1,522 | 1,522 | 1,522 |
| AIC | 9319.3 | 9319.8 | 9321.7 | 9322.4 |
| BIC | 9361.9 | 9367.7 | 9375.0 | 9386.3 |

Models with covariates plus: 1) no mobility variable; 2) any direction mobility variable; 3) variables to indicate upward or downward trend; 4) variables to indicate one step, two step upward or downward trend. Notes: 95% confidence intervals in brackets. * p < .05, ** p < .01, *** p < .001.

Table S13: DRM parameters estimates [95% confidence interval] for accelerated age indexed by DunedinPoAm for participants born before 1956

|  | Model 1 | Model 2 | Model 3 | Model 4 |
| --- | --- | --- | --- | --- |
| Disadvantage | 1.11^***^  [0.73,1.49] | 1.20^***^  [0.80,1.59] | 1.27^***^  [0.85,1.69] | 1.27^***^  [0.85,1.70] |
| Intermediate | -0.02  [-0.50,0.45] | 0.01  [-0.43,0.45] | -0.05  [-0.40,0.30] | -0.09  [-0.57,0.39] |
| Advantage | -1.09^***^  [-1.65,-0.52] | -1.21^***^  [-1.74,-0.68] | -1.22^***^  [-1.67,-0.77] | -1.18^***^  [-1.76,-0.60] |
| Origin occupation weight | 0.44  [0.19,0.70] | 0.41  [0.19,0.63] | 0.11  [-0.43,0.65] | 0.38  [-2.43,3.19] |
| Destination occupation weight | 0.56^***^  [0.30,0.81] | 0.59^***^  [0.37,0.81] | 0.89^**^  [0.35,1.43] | 0.62  [-2.19,3.43] |
| Female | -0.91^***^  [-1.32,-0.50] | -0.90^***^  [-1.31,-0.50] | -0.90^***^  [-1.31,-0.49] | -0.90^***^  [-1.31,-0.49] |
| Age | -0.16  [-0.39,0.07] | -0.17  [-0.40,0.06] | -0.17  [-0.40,0.06] | -0.17  [-0.40,0.06] |
| Age squared | 0.12  [-0.03,0.27] | 0.12  [-0.03,0.27] | 0.12  [-0.03,0.27] | 0.12  [-0.03,0.27] |
| Mobility in any direction |  | 0.47^*^  [0.02,0.92] |  |  |
| Downward mobility |  |  | -0.01  [-0.98,0.95] |  |
| Upward mobility |  |  | 0.98  [-0.01,1.97] |  |
| One-step downward mobility |  |  |  | 0.38  [-3.41,4.17] |
| Two-step downward mobility |  |  |  | 0.00  [-7.05,7.05] |
| One-step upward mobility |  |  |  | 0.66  [-2.76,4.08] |
| Two-step upward mobility |  |  |  | 0.21  [-6.92,7.33] |
| Constant | 0.26  [-0.10,0.62] | -0.04  [-0.49,0.41] | -0.04  [-0.49,0.40] | -0.03  [-0.49,0.42] |
| Observations | 1,522 | 1,522 | 1,522 | 1,522 |
| AIC | 8587.0 | 8584.7 | 8585.5 | 8589.0 |
| BIC | 8629.6 | 8632.7 | 8638.8 | 8652.9 |

Models with covariates plus: 1) no mobility variable; 2) any direction mobility variable; 3) variables to indicate upward or downward trend; 4) variables to indicate one step, two step upward or downward trend. Notes: 95% confidence intervals in brackets. * p < .05, ** p < .01, *** p < .001.

Table S14: DRM parameters estimates [95% confidence interval] for accelerated age indexed by Horvath for participants born after 1956 (1956 inclusive)

|  | Model 1 | Model 2 | Model 3 | Model 4 |
| --- | --- | --- | --- | --- |
| Disadvantage | -0.03  [-0.34,0.28] | -0.01  [-0.29,0.27] | -0.03  [-0.42,0.36] | -0.05  [-0.40,0.31] |
| Intermediate | 0.03  [-0.30,0.36] | 0.03  [-0.36,0.43] | 0.04  [-0.27,0.36] | 0.08  [-0.29,0.45] |
| Advantage | -0.00  [-0.32,0.32] | -0.02  [-0.24,0.19] | -0.01  [-0.32,0.30] | -0.03  [-0.40,0.33] |
| Origin occupation weight | 0.46  [-4.34,5.27] | -0.30  [-10.62,10.02] | -0.15  [-5.99,5.69] | 0.36  [-2.16,2.89] |
| Destination occupation weight | 0.54  [-4.27,5.34] | 1.30  [-9.02,11.62] | 1.15  [-4.69,6.99] | 0.64  [-1.89,3.16] |
| Female | -0.93^***^  [-1.24,-0.61] | -0.93^***^  [-1.25,-0.61] | -0.93^***^  [-1.25,-0.61] | -0.93^***^  [-1.24,-0.61] |
| Age | -0.11  [-0.28,0.06] | -0.11  [-0.28,0.06] | -0.11  [-0.28,0.06] | -0.11  [-0.28,0.06] |
| Age squared | -0.24^**^  [-0.39,-0.09] | -0.24^**^  [-0.39,-0.09] | -0.24^**^  [-0.39,-0.09] | -0.24^**^  [-0.39,-0.09] |
| Mobility in any direction |  | 0.13  [-0.26,0.52] |  |  |
| Downward mobility |  |  | 0.15  [-0.42,0.73] |  |
| Upward mobility |  |  | 0.11  [-0.39,0.61] |  |
| One-step downward mobility |  |  |  | 0.08  [-0.47,0.63] |
| Two-step downward mobility |  |  |  | 0.65  [-0.66,1.96] |
| One-step upward mobility |  |  |  | 0.09  [-0.34,0.52] |
| Two-step upward mobility |  |  |  | 0.18  [-0.47,0.82] |
| Constant | 0.78^***^  [0.48,1.08] | 0.71^***^  [0.34,1.08] | 0.71^***^  [0.34,1.08] | 0.70^***^  [0.33,1.07] |
| Observations | 1,618 | 1,618 | 1,618 | 1,618 |
| AIC | 8380.3 | 8381.9 | 8383.9 | 8387.1 |
| BIC | 8423.4 | 8430.4 | 8437.7 | 8451.8 |

Models with covariates plus: 1) no mobility variable; 2) any direction mobility variable; 3) variables to indicate upward or downward trend; 4) variables to indicate one step, two step upward or downward trend. Notes: 95% confidence intervals in brackets. * p < .05, ** p < .01, *** p < .001.

Table S15: DRM parameters estimates [95% confidence interval] for accelerated age indexed by Hannum for participants born after 1956 (1956 inclusive)

|  | Model 1 | Model 2 | Model 3 | Model 4 |
| --- | --- | --- | --- | --- |
| Disadvantage | 0.07  [-0.22,0.35] | 0.11  [-0.17,0.40] | 0.18  [-0.10,0.46] | 0.16  [-0.13,0.45] |
| Intermediate | -0.20  [-0.49,0.10] | -0.19  [-0.48,0.10] | -0.18  [-0.45,0.09] | -0.14  [-0.44,0.16] |
| Advantage | 0.13  [-0.10,0.35] | 0.08  [-0.30,0.45] | 0.00  [-0.28,0.29] | -0.02  [-0.32,0.28] |
| Origin occupation weight | 0.28  [-0.53,1.09] | 0.52  [-0.54,1.57] | 0.41  [-0.38,1.20] | 0.24  [-1.02,1.51] |
| Destination occupation weight | 0.72  [-0.09,1.53] | 0.48  [-0.57,1.54] | 0.59  [-0.20,1.38] | 0.76  [-0.51,2.02] |
| Female | -1.42^***^  [-1.68,-1.16] | -1.42^***^  [-1.68,-1.16] | -1.42^***^  [-1.68,-1.16] | -1.42^***^  [-1.68,-1.16] |
| Age | -0.05  [-0.19,0.09] | -0.06  [-0.20,0.09] | -0.06  [-0.20,0.08] | -0.06  [-0.20,0.08] |
| Age squared | -0.13^*^  [-0.26,-0.01] | -0.13^*^  [-0.26,-0.01] | -0.13^*^  [-0.26,-0.01] | -0.13^*^  [-0.26,-0.01] |
| Mobility in any direction |  | 0.15  [-0.18,0.48] |  |  |
| Downward mobility |  |  | -0.06  [-0.51,0.39] |  |
| Upward mobility |  |  | 0.23  [-0.08,0.54] |  |
| One-step downward mobility |  |  |  | -0.11  [-0.63,0.41] |
| Two-step downward mobility |  |  |  | 0.05  [-1.04,1.14] |
| One-step upward mobility |  |  |  | 0.20  [-0.15,0.54] |
| Two-step upward mobility |  |  |  | 0.37  [-0.26,0.99] |
| Constant | 0.95^***^  [0.68,1.22] | 0.89^***^  [0.58,1.19] | 0.88^***^  [0.58,1.19] | 0.88^***^  [0.58,1.18] |
| Observations | 1,618 | 1,618 | 1,618 | 1,618 |
| AIC | 7742.8 | 7744.1 | 7744.2 | 7747.9 |
| BIC | 7786.0 | 7792.6 | 7798.1 | 7812.5 |

Models with covariates plus: 1) no mobility variable; 2) any direction mobility variable; 3) variables to indicate upward or downward trend; 4) variables to indicate one step, two step upward or downward trend. Notes: 95% confidence intervals in brackets. * p < .05, ** p < .01, *** p < .001.

Table S16: DRM parameters estimates [95% confidence interval] for accelerated age indexed by Phenoage for participants born after 1956 (1956 inclusive)

|  | Model 1 | Model 2 | Model 3 | Model 4 |
| --- | --- | --- | --- | --- |
| Disadvantage | 0.88^***^  [0.46,1.30] | 0.89^***^  [0.47,1.32] | 0.82^***^  [0.34,1.30] | 0.83^***^  [0.34,1.31] |
| Intermediate | -0.55^*^  [-1.00,-0.09] | -0.54^*^  [-0.99,-0.09] | -0.54^*^  [-1.00,-0.07] | -0.55^*^  [-1.05,-0.04] |
| Advantage | -0.33  [-0.76,0.10] | -0.35  [-0.79,0.09] | -0.28  [-0.78,0.21] | -0.28  [-0.79,0.23] |
| Origin occupation weight | 0.43  [0.14,0.72] | 0.44  [0.15,0.72] | 0.54  [0.09,0.99] | 0.56  [0.06,1.06] |
| Destination occupation weight | 0.57^***^  [0.28,0.86] | 0.56^***^  [0.28,0.85] | 0.46^*^  [0.01,0.91] | 0.44  [-0.06,0.94] |
| Female | 0.14  [-0.30,0.58] | 0.14  [-0.30,0.58] | 0.14  [-0.30,0.57] | 0.14  [-0.30,0.57] |
| Age | -0.20  [-0.44,0.04] | -0.20  [-0.44,0.03] | -0.20  [-0.44,0.04] | -0.20  [-0.44,0.04] |
| Age squared | -0.40^***^  [-0.61,-0.19] | -0.40^***^  [-0.61,-0.19] | -0.40^***^  [-0.61,-0.19] | -0.40^***^  [-0.61,-0.19] |
| Mobility in any direction |  | 0.13  [-0.33,0.60] |  |  |
| Downward mobility |  |  | 0.36  [-0.46,1.18] |  |
| Upward mobility |  |  | 0.05  [-0.46,0.56] |  |
| One-step downward mobility |  |  |  | 0.38  [-0.50,1.26] |
| Two-step downward mobility |  |  |  | 0.37  [-1.52,2.27] |
| One-step upward mobility |  |  |  | 0.06  [-0.47,0.60] |
| Two-step upward mobility |  |  |  | -0.01  [-1.02,1.00] |
| Constant | 0.42^*^  [0.02,0.82] | 0.33  [-0.18,0.84] | 0.34  [-0.17,0.85] | 0.34  [-0.17,0.85] |
| Observations | 1,618 | 1,618 | 1,618 | 1,618 |
| AIC | 9419.1 | 9420.8 | 9422.4 | 9426.3 |
| BIC | 9462.2 | 9469.3 | 9476.3 | 9491.0 |

Models with covariates plus: 1) no mobility variable; 2) any direction mobility variable; 3) variables to indicate upward or downward trend; 4) variables to indicate one step, two step upward or downward trend. Notes: 95% confidence intervals in brackets. * p < .05, ** p < .01, *** p < .001.

Table S17: DRM parameters estimates [95% confidence interval] for accelerated age indexed by DunedinPoAm for participants born after 1956 (1956 inclusive)

|  | Model 1 | Model 2 | Model 3 | Model 4 |
| --- | --- | --- | --- | --- |
| Disadvantage | 1.02^***^  [0.73,1.30] | 1.01^***^  [0.73,1.30] | 1.03^***^  [0.72,1.34] | 1.06^***^  [0.74,1.37] |
| Intermediate | -0.21  [-0.50,0.08] | -0.22  [-0.51,0.08] | -0.21  [-0.50,0.08] | -0.32  [-0.64,0.01] |
| Advantage | -0.81^***^  [-1.09,-0.52] | -0.80^***^  [-1.09,-0.51] | -0.82^***^  [-1.13,-0.50] | -0.74^***^  [-1.07,-0.41] |
| Origin occupation weight | 0.35  [0.17,0.53] | 0.35  [0.17,0.53] | 0.30  [-0.10,0.70] | 0.62  [0.04,1.19] |
| Destination occupation weight | 0.65^***^  [0.47,0.83] | 0.65^***^  [0.47,0.83] | 0.70^***^  [0.30,1.10] | 0.38  [-0.19,0.96] |
| Female | -0.49^***^  [-0.77,-0.20] | -0.49^***^  [-0.77,-0.20] | -0.48^***^  [-0.77,-0.20] | -0.49^***^  [-0.77,-0.20] |
| Age | -0.04  [-0.20,0.11] | -0.04  [-0.20,0.11] | -0.04  [-0.20,0.11] | -0.04  [-0.20,0.11] |
| Age squared | -0.02  [-0.16,0.12] | -0.02  [-0.16,0.12] | -0.02  [-0.16,0.12] | -0.02  [-0.16,0.12] |
| Mobility in any direction |  | -0.06  [-0.37,0.24] |  |  |
| Downward mobility |  |  | -0.15  [-0.81,0.52] |  |
| Upward mobility |  |  | 0.00  [-0.54,0.54] |  |
| One-step downward mobility |  |  |  | 0.28  [-0.55,1.10] |
| Two-step downward mobility |  |  |  | 0.09  [-1.49,1.67] |
| One-step upward mobility |  |  |  | -0.17  [-0.67,0.32] |
| Two-step upward mobility |  |  |  | -0.78  [-1.91,0.35] |
| Constant | 0.42^**^  [0.16,0.68] | 0.46^**^  [0.13,0.79] | 0.46^**^  [0.13,0.79] | 0.47^**^  [0.14,0.80] |
| Observations | 1,618 | 1,618 | 1,618 | 1,618 |
| AIC | 8021.6 | 8023.4 | 8025.3 | 8027.5 |
| BIC | 8064.7 | 8071.9 | 8079.2 | 8092.2 |

Models with covariates plus: 1) no mobility variable; 2) any direction mobility variable; 3) variables to indicate upward or downward trend; 4) variables to indicate one step, two step upward or downward trend. Notes: 95% confidence intervals in brackets. * p < .05, ** p < .01, *** p < .001.
